# *Trichomonas vaginalis* Legumain-2, TvLEGU-2, Is an Immunogenic Cysteine Peptidase Expressed during Trichomonal Infection

**DOI:** 10.3390/pathogens13020119

**Published:** 2024-01-27

**Authors:** Esly Alejandra Euceda-Padilla, Miriam Guadalupe Mateo-Cruz, Leticia Ávila-González, Claudia Ivonne Flores-Pucheta, Jaime Ortega-López, Daniel Talamás-Lara, Beatriz Velazquez-Valassi, Lidia Jasso-Villazul, Rossana Arroyo

**Affiliations:** 1 Departamento de Infectómica y Patogénesis Molecular, Centro de Investigación y de Estudios Avanzados del Instituto Politécnico Nacional (CINVESTAV-IPN), Av. IPN 2508, Alcaldía Gustavo A. Madero (GAM), Mexico City 07360, Mexico; esly.euceda@cinvestav.mx (E.A.E.-P.); miriam.mateo@cinvestav.mx (M.G.M.-C.); lavilag@cinvestav.mx (L.Á.-G.); 2 Departamento de Biotecnología y Bioingeniería, Centro de Investigación y de Estudios Avanzados del Instituto Politécnico Nacional (CINVESTAV-IPN), Av. IPN 2508, Alcaldía Gustavo A. Madero (GAM), Mexico City 07360, Mexico; cflores@cinvestav.mx (C.I.F.-P.); jortega@cinvestav.mx (J.O.-L.); 3 Unidad de Microscopía Electrónica, Laboratorios Nacionales De Servicios Experimentales (LaNSE), Centro de Investigación y de Estudios Avanzados del Instituto Politécnico Nacional (CINVESTAV-IPN), Av. IPN 2508, Alcaldía Gustavo A. Madero (GAM), Mexico City 07360, Mexico; dtalamas@cinvestav.mx; 4 Departamento de Vigilancia Epidemiológica, Hospital General de México “Eduardo Liceaga”, Mexico City 06720, Mexico; betvelval@gmail.com; 5 Unidad de Medicina Preventiva, Hospital General de México “Eduardo Liceaga”, Mexico City 06720, Mexico; jasso.misty@gmail.com

**Keywords:** legumain-like cysteine peptidase, immunogenic peptidase, glucose, *Trichomonas vaginalis*, TvLEGU-2, vaginal secretions

## Abstract

*Trichomonas vaginalis* is the causative agent of trichomoniasis, the most prevalent nonviral, neglected sexually transmitted disease worldwide. *T. vaginalis* has one of the largest degradomes among unicellular parasites. Cysteine peptidases (CPs) are the most abundant peptidases, constituting 50% of the degradome. Some CPs are virulence factors recognized by antibodies in trichomoniasis patient sera, and a few are found in vaginal secretions that show fluctuations in glucose concentrations during infection. The CPs of clan CD in *T. vaginalis* include 10 genes encoding legumain-like peptidases of the C13 family. TvLEGU-2 is one of them and has been identified in multiple proteomes, including the immunoproteome obtained with Tv (+) patient sera. Thus, our goals were to assess the effect of glucose on TvLEGU-2 expression, localization, and in vitro secretion and determine whether TvLEGU-2 is expressed during trichomonal infection. We performed qRT-PCR assays using parasites grown under different glucose conditions. We also generated a specific anti-TvLEGU-2 antibody against a synthetic peptide of the most divergent region of this CP and used it in Western blot (WB) and immunolocalization assays. Additionally, we cloned and expressed the *tvlegu-2* gene (TVAG_385340), purified the recombinant TvLEGU-2 protein, and used it as an antigen for immunogenicity assays to test human sera from patients with vaginitis. Our results show that glucose does not affect *tvlegu-2* expression but does affect localization in different parasite organelles, such as the plasma membrane, Golgi complex, hydrogenosomes, lysosomes, and secretion vesicles. TvLEGU-2 is secreted in vitro, is present in vaginal secretions, and is immunogenic in sera from Tv (+) patients, suggesting its relevance during trichomonal infection.

## 1. Introduction

Trichomoniasis is a neglected sexually transmitted infection (STI) caused by the protozoan parasite *Trichomonas vaginalis.* It is the most prevalent nonviral STI worldwide, with approximately ∼110 million new cases annually, according to the WHO [1]. Symptomatic patients present frothy, yellow, or green, mucopurulent, and foul-smelling discharge along with dysuria and pruritus [2]. Complications associated with trichomoniasis include preterm labor, infertility, and an increased risk of cervical and prostatic cancer [3,4,5,6,7].

The *T. vaginalis* genome contains ∼440 genes that encode peptidases, half of which are cysteine proteinases (CPs). Forty-eight are cathepsin L-like and 10 are legumain-like CPs [8], but less than half show proteolytic activity by two-dimensional gel electrophoresis (2-DE) zymography [9,10,11]. CPs are virulence factors involved in the pathogenesis of *T. vaginalis* [12]. Some CPs are highly immunogenic and are also found in the secretions of infected patients [13,14]. In the extensive *T. vaginalis* degradome, only nine CPs have been identified by mass spectrometry (MS): seven cathepsin L-like CPs (TvCP1, TvCP2, TvCP3, TvCP4, TvCP4-like, TvCP12, and TvCP39) and two asparaginyl endopeptidases of the C13 family (TvLEGU-1 and TvLEGU-2) [14,15,16]. These CPs have been identified and characterized as important virulence factors [15,16] and participate in cytotoxicity (TvCP65, TvCP39, TvCP12, and TvCP2) [17,18,19,20], hemolysis (TvCP4) [11], and cytoadherence (TvLEGU-1 and CP30) [21,22]. These CPs are also highly regulated by environmental factors, such as pH, iron, glucose, zinc, and polyamines. All characterized CPs in *T. vaginalis* belong to clans CA and CD, and the C1 family (CA clan), which is reported to have the highest activity in this parasite, contains most of the proteinases. The CPs of family C13 (clan CD) are also important in *T. vaginalis* biology [16].

The *T. vaginalis* CP genes of family C13 include 10 genes encoding legumain-like CPs [8]. After the genome was sequenced, there was a reclassification of the parasite genes encoding legumain proteins. Thus, the gene reported as *tvlegu-2* by León-Félix et al. [23] corresponds to the current *tvlegu-9* (TVAG_305110). The actual *tvlegu-2* gene (TVAG_385340) has been identified in multiple proteomes, such as the immunodegradome of sera from patients with trichomoniasis [10], secretion products [24,25], extracellular vesicles [26], and lysosomes [27]. Interestingly, TvLEGU-2 appears to be differentially regulated by iron and glucose (Cárdenas-Guerra et al., unpublished data [20,28,29].

Here, we analyzed the effect of glucose on the amounts of TvLEGU-2 transcript, protein, and protein localization. In addition, we confirmed its expression during infection using samples of sera and vaginal washes (VWs) from trichomoniasis patients to demonstrate its immunogenicity and presence in vaginal secretions.

## 2. Materials and Methods

### 2.1. Bioinformatics Analysis of tvlegu-2 and TvLEGU-2

The 5′ and 3′ UTR regions of the *tvlegu-2* gene in the *T. vaginalis* database with accession number TVAG_385340 [8] (https://trichdb.org/trichdb/app) (accessed on 20 April 2020) were analyzed. The deduced amino acid sequence of TvLEGU-2 was used to predict posttranslational modifications (PTMs) using the TMHMM Server v. 2.0 (http://www.cbs.dtu.dk/services/TMHMM/) (accessed on 20 April 2020), Prosite (https://prosite.expasy.org/) (accessed on 21 April 2020), SecretomeP 2.0 Server (http://www.cbs.dtu.dk/services/SecretomeP/) (accessed on 21 April 2020), TargetP-2.0 Server (http://www.cbs.dtu.dk/services/TargetP/) (accessed on 21 April 2020), Cell-Ploc 2.0 (http://www.csbio.sjtu.edu.cn/bioinf/Cell-PLoc-2/) (accessed on 21 April 2020), and CELLO v. 2.5 (http://cello.life.nctu.edu.tw/) (accessed on 21 April 2020) platforms. Protein domain analysis was performed using the Interpro Server (https://www.ebi.ac.uk/interpro/) (accessed on 21 April 2020). The AlphaFold Protein Structure Database (https://alphafold.ebi.ac.uk/) (accessed on 31 May 2022) was used to predict the three-dimensional (3D) model of the native TvLEGU-2 structure created with the AlphaFold Monomer v2.0 pipeline (31 May 2022). The models were visualized with UCSF Chimera 1.16 software.

### 2.2. Trichomonas Vaginalis In Vitro Cultures

The CNCD 188 *T. vaginalis* isolate was subjected to one week of in vitro culture and used in this study. Parasites were grown at 37 °C in tryptone-yeast extract (TY) medium supplemented with 10% heat-inactivated adult bovine serum (HIBS) and glucose (D(+)-glucose anhydrous; Cat. No. 15866, Merck, Darmstatd, Germany) to a final concentration of 25 mM (normal glucose; NG). Assays were initiated with cultures in 50 mM glucose (high glucose; HG) or without glucose addition <1 mM (glucose restriction; GR) TY medium with 10% HIBS, as previously reported [30].

### 2.3. qPCR

Total RNA was extracted from parasites grown in different glucose culture conditions (GR and HG) using TRIzol (Thermo Scientific-Pierce, Rockford, IL, USA). For cDNA synthesis, SCRIPT cDNA Synthesis Kit (Jena Bioscience, Jena, Germany) reverse transcriptase was used, following the manufacturer’s instructions. For qPCR, a qPCR SybrMaster highROX kit (Jena Bioscience) was used. A 100 ng aliquot of cDNA from GR or HG conditions was used as a template, and a 162 bp fragment (536–697 bp) of the *tvlegu-2* gene (TVAG_385340) was amplified in a reaction mixture (20 µL) containing 10 µL of qPCR SybrMaster reaction mix and 10 µM of each specific primer for the *tvlegu-2* gene: sense primer *tvlegu-2* F (5′-AGGTTGTTGTTGCTGGTGTTCCA-3′) and anti-sense primer *tvlegu-2* R (5′-CGATTGTGCATGTTGGGTGG-3′). The cycling conditions used were as follows: 95 °C for 2 min, followed by 40 cycles of 95 °C for 15 s and 60 °C for 1 min on the Applied Biosystems™ StepOne™ Real-Time PCR System (Thermo Fisher Scientific, Waltham, MA, USA) (kindly lent by Dr. Fidel Hernández-Hernández). The *α-tubulin* (TVAG_206890) gene was used to normalize the results, and a 101 bp transcript was amplified using the following specific primers: sense primer *α-tubulin* F (5′-TGCCCAACAGGCTTCAAGAT-3′) and anti-sense primer *α-tubulin* R (5′-TTAGCGAGCATGCAGACAGC-3′) [31]. The data were analyzed in GraphPad Prism 8.0.1. Three independent assays were performed in duplicate with similar results.

### 2.4. Cloning and Expression of TvLEGU-2r

The *tvlegu-2* gene (1179 bp) (GenBank XP_001303267.1; TVAG_385340) was synthesized with sites for the restriction enzymes *Nco*I and *Xho*I in the 5′ and 3′ regions, respectively, and without the signal peptide (1145 bp) (Synbio Technologies, Monmouth Junction, NJ, USA). The construct was subcloned and inserted into the pCri1a prokaryotic expression vector [32]. Expression of the TvLEGU-2 recombinant (TvLEGU-2r) protein was induced in *E. coli* BL21 (DE3) with 0.1 mM isopropyl-β-D-1-thiogalactopyranoside (IPTG) for 14 h at 20 °C and 250 rpm. TvLEGU-2r protein expression was visualized by electrophoresis under denaturing conditions (SDS–PAGE) on 12% polyacrylamide gels and by WB assays using an α-6xHis Tag monoclonal antibody (Sigma-Aldrich, San Luis, MO, USA). The TvLEGU-2r protein from inclusion bodies was solubilized and purified by affinity chromatography using Ni-Sepharose 6 Fast Flow columns (GE Healthcare—Amersham Biosciences, Amersham, UK), following the instructions of the manufacturer.

### 2.5. Production of α-TvLEGU-2 and α-CNCD188 Antibodies

To produce an anti-TvLEGU-2 peptide or an anti-TvLEGU-2r antibody, two groups of animals were used. In the first group, the TvLEGU-2-specific synthetic peptide QSHVMEYGDTSLK conjugated with the KHL carrier (PepMic, Suzhou, China) was used as an antigen to inoculate 15,6 week old female BALB/c mice and 1,8 week old 1.5 kg male New Zealand rabbit. In the second group, the recombinant TvLEGU-2 (TvLEGU-2r) protein was used as an antigen to inoculate 15,6 week old female BALB/c mice and 1,8 week old 1.5 kg male New Zealand rabbit. Mice were immunized by subcutaneous and intramuscular injections of 50 µg of the synthetic peptide, or TvLEGU-2r, as an antigen with TiterMax gold as an adjuvant. Rabbits were immunized via intramuscular injection of 300 µg of the synthetic peptide, or TvLEGU-2r, as an antigen with TiterMax gold as an adjuvant. The resulting antiserum (Mα-TvLEGU-2pep or Rα-TvLEGU-2pep) was used in Western blot (WB) and indirect immunofluorescence assays. To produce an anti-trichomonad antibody, parasites (2 × 10^7^) from the CNCD188 trichomonad isolate were lysed by three freeze and thaw cycles. Tv lysate emulsified with Freund’s complete adjuvant (Gibco™, Waltham, MA, USA) was used as an antigen to immunize a male rabbit by intramuscular injection. The animal was given booster injections three times with the same antigen in Freund’s incomplete adjuvant (Gibco) at 3 week intervals [17]. This antiserum (anti-CNCD188) was used in indirect immunofluorescence assays. Preimmune (PI) serum from each animal was obtained before starting the immunization schedule, checked by WB against total trichomonad extracts (Supplementary Figure S5), and used as a negative control in all the experiments with antibodies [17]. We also used a secondary antibody alone as an extra-negative control. Both types of negative controls gave no reaction in WB assays or immunolocalization assays, as expected.

### 2.6. Protein Extracts for 1-D and 2-D Gel Electrophoresis (1-DE and 2-DE)

To obtain proteins present in in vitro secretion supernatants or vaginal washes (VWs) from Tv (+) and Tv (−) patients, samples were precipitated with 10% trichloroacetic acid (TCA) at 4 °C overnight [20]. Proteins were separated by SDS–PAGE on a 10% polyacrylamide gel. To obtain protease-resistant extracts (PRE) for 1-DE, 8 × 10^6^ parasites were lysed directly with PBS pH 8.0 and 10% sodium deoxycholate (DOC) without protease inhibitors for 20 min at 4 °C and centrifuged at 13,000× *g* for 30 min at 4 °C using a 10% sucrose cushion. The supernatant was recovered, and the protein concentration was quantified by NanoDrop. For 2-DE assays, PRE was obtained from parasites (6 × 10^7^) lysed directly in 2-DE rehydration buffer (Bio-Rad, Hercules, CA, USA) in the absence of proteinase inhibitors. After 20 min of incubation at 25 °C, cell debris was removed by centrifugation at 13,000× *g* for 10 min at 4 °C. The supernatant was recovered, and the protein samples were subjected to isoelectric focusing (IEF) using ReadyStrip strips with an immobilized pH gradient (IPG) (7 cm, linear pH 4–7; Bio-Rad). The samples were actively hydrated at 2500 V for 16 h at 22 °C in the Protean IEF Cell (Bio-Rad) in three steps: 250 V for 20 min, 4000 V for 3 h, and a gradual increase from 4000 V to 10,000 V/h. After reduction and alkylation in the equilibration buffer (Bio-Rad), the IPG strips were subjected to 2-DE on polyacrylamide gels. The gels were stained with Coomassie Brilliant Blue or silver stain for protein detection or were transferred onto a 0.22 μm nitrocellulose (NC) membrane for WB analysis with different antibodies [10].

### 2.7. Western Blot

Proteins from PRE, purified TvLEGU-2r, proteins from in vitro secretions, or VWs were transferred onto 0.22 μm NC membranes (Bio-Rad) for WB assays. The membranes used for in vitro secretion samples were incubated with the primary antibody Rα-TvLEGU-2pep (1:1000) antibody or with the PI serum or secondary antibody alone as a negative control for 18 h at 4 °C, washed with PBS-Tween 0.1%, incubated with a peroxidase-conjugated polyclonal goat α-rabbit secondary antibody (1:5000 dilution; Invitrogen, Waltham, MA, USA) for 1 h at 37 °C, and developed with an enhanced chemiluminescence system (SuperSignal Chemiluminescent Substrate West Pico; Thermo Scientific-Pierce, Rockford, IL, USA) on a Chemidoc XR (Bio-Rad). For immunogenicity assays, sera from Tv (+) and Tv (−) patients were used as primary antibody solutions, and TvLEGU-2r on NC membranes was used as an antigen. To evaluate the cross-reactivity of the Rα-TvLEGU-2pep antibody with TvLEGU-1 or the Rα-TvLEGU-1r antibody [22] with TvLEGU-2r, WB assays were performed using NC membranes containing TvLEGU-1r or TvLEGU-2r protein as antigens and incubated with the following primary antibodies: one set of both recombinant proteins (TvLEGU-1r or TvLEGU-2r) was incubated with the Rα-TvLEGU-1r antibody, and the second set of NC membranes was incubated with the Rα-TvLEGU-2pep antibody. For all assays, ImageLab software 5.0 (Bio-Rad) was used for molecular weight analysis.

### 2.8. TvLEGU-2 Localization by Indirect Immunofluorescence Assays (IFA)

Parasites cultured under different glucose conditions were fixed with 4% formaldehyde in PBS for 30 min at room temperature (RT) on poly-L-lysine-treated slides (Sigma-Aldrich). Nonpermeabilized parasites were washed, blocked with 20 mM NH_4_Cl in PBS (NH_4_Cl/PBS) and 0.2% bovine serum albumin (BSA) in PBS (BSA/PBS), and incubated with primary antibodies (Mα-TvLEGU-2pep and Rα-CNCD 188 antibody at 1:100 and 1:200 dilution, respectively) or with the PI serum or secondary antibody alone as negative controls for 2 h at RT. The specificity of the Mα-TvLEGU-2pep antibody against TvLEGU-2 was checked by using the 2-DE-WB assay (Supplementary Figure S6A) and IFA (Supplementary Figure S6B). In both types of assays, the mouse antibody gave identical recognition of TvLEGU-2 as the rabbit antibody generated against the synthetic peptide. The samples were then washed with BSA/PBS, incubated with Alexa-Fluor 647-conjugated goat α-mouse (dilution 1:300) and fluorescein isothiocyanate (FITC; 1:100 dilution)-conjugated goat α-rabbit (Thermo-Scientific-Pierce, USA) secondary antibodies for 1 h at room temperature (RT), and washed with BSA/PBS and PBS. For intracellular labeling, parasites were permeabilized with 0.2% Triton X-100 for 10 min and treated as previously described. For TvLEGU-2/PFO and TvLEGU-2/TvAtg8 double-labeling assays, the above protocol was performed by incubation with the primary antibodies: Mα-TvLEGU-2pep and Rα-TvPFO50r, an antibody generated against a recombinant 50 kDa COOH-terminal fragment of the pyruvate ferredoxin:oxidoreductase a (PFOa) trichomonad protein previously characterized [33]; Mα-TvLEGU-2pep and Rα-rTvAtg8b, an antibody generated against a recombinant TvAtg8b autophagic protein previously characterized [34] at 1:100 and 1:50 dilution, respectively, or with the PI serum or secondary antibody alone as a negative control for 2 h at RT, followed by washing with BSA/PBS and PBS. All slides were incubated with 4′,6-diamidino-2-phenylindole (DAPI)/PBS for 15 min at RT and washed with PBS. Samples were mounted with a Vectashield mounting solution and analyzed by confocal microscopy and ZEISS Efficient Navigation (ZEN) software 3.8 (Zeiss, Oberkochen, Germany).

For TvLEGU-2 colocalization with lysosomes, parasites were cultured for 12 h with 5 μM LysoTracker Red DND-99 (Invitrogen) in TY-HIBS medium at 37 °C, washed with PBS, fixed with 4% formaldehyde for 1 h at 37 °C, and washed again with Hanks Balanced Salt Solution (HBSS) (Gibco). Parasites were permeabilized with 0.1% Triton X-100 for 10 min, washed with HBSS/BSA, blocked with 0.5 M glycine for 1 h at RT, and washed again with HBSS/BSA. The parasites were then processed for an indirect immunofluorescence assay with the Mα-TvLEGU-2pep antibody, as described above.

### 2.9. Transmission Electron Microscopy Immunolabeling

Parasites (2 × 10^7^) grown under different glucose conditions were fixed in 2.5% glutaraldehyde in 0.1 M sodium cacodylate buffer at pH 7.2 at RT, treated with 1% (*w*/*v*) osmium tetroxide, and dehydrated with increasing concentrations of ethanol. Samples were transferred to propylene oxide and then successively to propylene oxide/LR-White resin mixtures at ratios of 1:1 and 2:1, embedded in LR-White resin, and polymerized at 56 °C overnight. Thin sections (60 nm) were obtained and incubated overnight at RT with the Mα-TvLEGU-2pep (mouse, 1:10) primary antibody, washed, and incubated with gold-conjugated anti-mouse IgG (1:60 dilution using 15 nm gold particles) as a secondary antibody (Ted Pella Inc., Redding, CA, USA). For double labeling, the sections were incubated with Mα-TvLEGU-2pep/RαTvPFO50r and Mα-TvLEGU-2pep/RαrTvAtg8b primary antibody combinations (both at 1:10 dilution). Gold-conjugated anti-mouse IgG (1:60 dilution; 15 nm gold particles) and anti-rabbit IgG (1:60 dilution; 30 nm gold particles) were used as secondary antibodies (Ted Pella Inc.). The samples were counterstained with uranyl acetate and lead citrate. Samples incubated with PI serum or only the secondary antibodies were used as negative controls. A JEOL JEM-1011 transmission electron microscope was used (JEOL Ltd., Tokyo, Japan).

### 2.10. In Vitro Secretion Assay

Parasites (2 × 10^7^) were resuspended in warm PBS at pH 7 supplemented with a final concentration of 0 or 50 mM glucose (GR and HG, respectively) at a parasite density of 4 × 10^6^/mL and allowed to perform secretion at 37 °C for 1 h. Cell viability was checked by trypan blue exclusion at the end of the assay. Samples were centrifuged at 1800× *g* to eliminate parasites, the supernatant was separated in a new conical tube and precipitated with 10% TCA at 4 °C overnight, and the presence of TvLEGU-2 was evaluated by WB assays.

### 2.11. Statistical Analysis

Statistically significant differences between means were determined by analysis of variance and the Student’s *t*-test using GraphPad Prism 8.0.1. The scores with statistical significance are indicated by asterisks in the figures. The corresponding *p* values are indicated in the figure legends.

## 3. Results

### 3.1. In Silico Analysis of the tvlegu-2 Gene and Deduced Amino Acid Sequence of the TvLEGU-2 Protein

**Figure 1:**
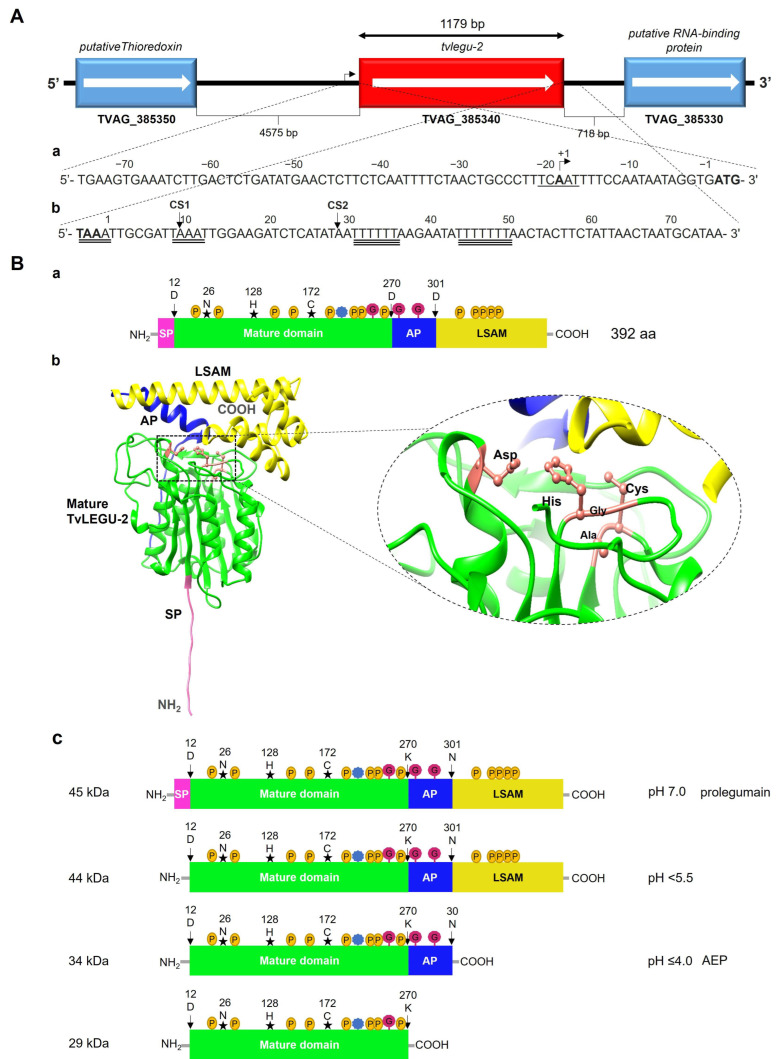
In silico analysis of the *tvlegu-2* gene and amino acid sequences. (**A**) Diagram of the *tvlegu-2* gene (TVAG_385340) in contig DS114117. Red: *tvlegu-2* gene with a size of 1179 bp; light blue: flanking genes encoding a putative thioredoxin (TVAG_385350) and a putative RNA-binding protein (TVAG_385330). The 5′ intergenic region is 4575 nt. (**Aa**) The 5′ regulatory region was analyzed and contains an Inr consensus sequence for transcription initiation (underlined); +1 indicates the possible transcription start site (black bend arrow). (**Ab**) The 3′ intergenic region is 718 nt. The 3′ downstream region contains two probable polyadenylation sites (PS_1_ and PS_2_, double underlining), two probable cleavage sites (CS_1_ and CS_2_; black arrow), and two downstream element sequences (DSE_1_ and DSE_2_; triple underline). (**B**) Structure and putative posttranslational modifications of the 392 aa precursor TvLEGU-2 protein. Pink: signal peptide (SP), green: catalytic domain (CD), blue: activation peptide (AP), and yellow: C-terminal domain (LSAM). (**Ba**) Yellow circles correspond to predicted phosphorylation sites; pink hexagon corresponds to a putative glycosylation site with a score >0.7; TRAF6 motif (blue star, 32 picks); catalytic residues: Asn (N)/His (H)/Cys (**c**) (black star); cleavage sites (black arrows). (**Bb**) Theoretical three-dimensional (3-D) model of TvLEGU-2 in AlphaFold2 v1.5.2. The 3-D model of TvLEGU-2 pro-peptidase, location of the His/Cys (His-Gly-X-Ala-Cys) catalytic dyad (close-up). (**Bc**) Putative processing steps of TvLEGU-2. Inactive proenzyme is synthesized at a neutral pH and undergoes autocatalytic processing at the C-terminal Asn^301^ (pH < 5.5) and K^270^ (pH ≤ 4.0) sites and N-terminally after Cys^11^. The N-terminal processing is not essential for enzyme activation, but the release of the C-terminal LSAM domain is essential to gaining full AEP activity.

The 1179 bp *tvlegu-2* gene (TVAG_385340) without introns is in contig DS114117 at 6741 to 7919 on the antisense strand (−). It is flanked by putative thioredoxin-encoding and RNA-binding protein-encoding genes (Figure 1A). The 5′ intergenic region at the upstream end of the *tvlegu-2* ORF is 4575 bp long. An Inr transcription promoter element (TCAAT) [35] is located 17 bp upstream from the ATG start codon (Figure 1(Aa)). The 3′ intergenic region is 718 bp long. In the downstream region of the stop codon, two putative polyadenylation signals (PSs) containing the tetranucleotide TAAA are found. PS_1_ overlaps the stop codon. PS_2_ is located +9 bp downstream of the stop codon. Two putative Py(A)_0–3_TTAA cleavage sites (CSs) are also present. The CS_1_ is +8 bp downstream of PS_1_, and the CS_2_ is +16 bp downstream of PS_2_. Additionally, there are two T-rich regions dubbed downstream elements (DSEs). DSE_1_ is +21 bp downstream of CS_1_, and DSE_2_ is +16 bp downstream of CS_2_ [36] (Figure 1(Aa,Ab)).

The complete *tvlegu-2* gene was deduced to encode a 392 amino acid inactive proenzyme or zymogen, which is named TvLEGU-2. Computational analysis suggests the presence of posttranslational modifications, such as 13 putative phosphorylation sites (score > 0.8), 8 of which are inside the catalytic domain, 2 putative O-glycosylation sites (score > 0.8) outside the catalytic domain, and one putative acetylation site in the signal peptide region (Figure 1(Ba)). TvLEGU-2 starts with a signal peptide of 11 amino acids (SP; Met^1^-Cis^11^), followed by the peptidase domain (Asp^12^-Lys^270^) of 259 amino acids and a C-terminal prodomain (Val^271^-Leu^392^) of 122 amino acids (Figure 1(Ba)). This organization of TvLEGU-2 is different from that of human legumain (HsLegumain), although HsLegumain also contains a short eight-amino acid sequence in the N-terminal propeptide [37]. In the 3D theoretical model of TvLEGU-2, the peptidase domain retains the caspase-equivalent topology, similar to that of the mammalian C13 family with five α-helices and six β-sheets [38] (Figure 1(Bb)). It also contains the catalytic triad His^128^/Cys^172^/Asn^26^ (Figure 1B, b zoom), which is characteristic of the C13 family peptidases, and the -His-Gly-[space]-Ala-Cys- motif of the catalytic site [39]. The structure of the proenzyme reveals that the C-terminal domain has two parts: an activation peptide (AP) (Val^271^-Asn^300^) and the legumain stabilization and activity modulation (LSAM) domain (Asn^301^-Leu^392^) that adopts a death-domain-like architecture typical in caspases and paracaspases [38], which shows some differences. In mammals, the LSAM domain is made up of five α-helices, but TvLEGU-2 contains only four α-helices (Figure 1(Bb)).

Based on the reported processing of HsLegumain [40], we hypothesized that the TvLEGU-2 processing steps would occur as follows: The TvLEGU-2 zymogen (∼45 kDa and a theoretical *pI* of 6.37) during endolysosomal trafficking is exposed to a change from basic to acidic pH that induces stepwise self-processing of the SP, LSAM, and AP domains, producing peptidase intermediate species of ∼44 and ∼34 kDa and mature peptidase of ∼29 kDa, respectively (Figure 1(Bc)).

### 3.2. Glucose Conditions Have No Impact on tvlegu-2 Gene Expression

**Figure 2:**
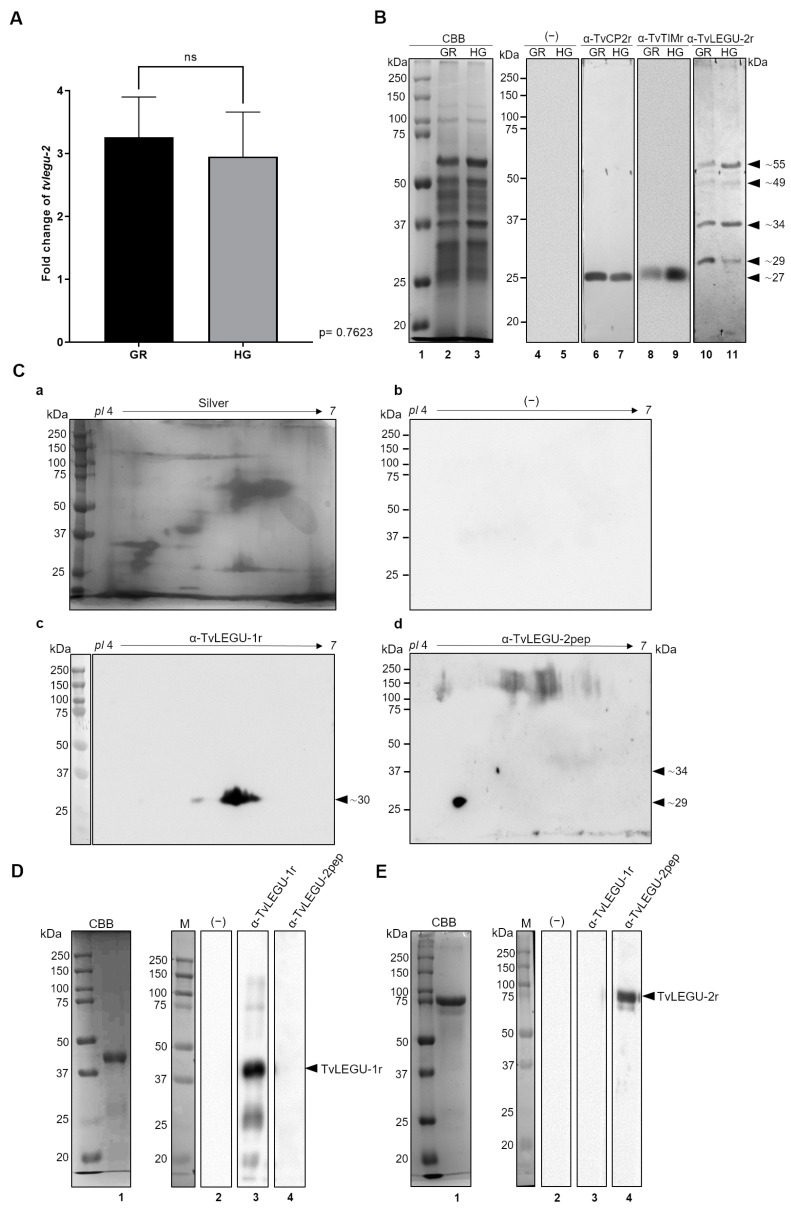
Effect of glucose on the mRNA and protein expression of TvLEGU-2. (**A**) qPCR with specific primers for the *tvlegu-2* and *α-tubulin* genes using 100 ng of cDNA from parasites grown in glucose restriction (GR) and high-glucose (HG) conditions. *α-tubulin* was used as a normalizing gene; ns, no significant differences. (**B**) Western blot assay of PREs from parasites grown under GR (<1 mM) and HG (50 mM). Coomassie Brilliant-Blue (CBB)-stained 10% SDS PAGE gel for PREs from parasites grown under GR and HG conditions (Lanes 2 and 3), respectively. For WB assays, duplicate gels were transferred onto NC membranes and incubated with different antibodies: Rα-TvLEGU-2pep (1:500 dilution) to detect the TvLEGU-2 protein, Rα-TvCP2r (1:6000 dilution) to detect a control protein overexpressed under GR conditions, Rα-TvTIM (1:1000 dilution) to detect an overexpressed TvTIM control protein under HG conditions, and a negative control with PI serum or no primary antibody (−). Arrowheads show the position of the native TvLEGU-2 (∼55, ∼49, ∼34, and ∼29 kDa) protein bands. (**C**) Silver-stained 2-DE protease-rich extracts from parasites grown in normal glucose conditions (25 mM) (**Ca**). WB of duplicate gels transferred onto NC membranes incubated with Rα-TvLEGU-1r (1:1000 dilution) antibody (**Cc**) to serve as a control for the specificity of Rα-TvLEGU-2pep antibody against other legumain proteins, Rα-TvLEGU-2pep (1:100 dilution) (**Cd**) to detect the TvLEGU-2 protein in PREs, or a negative control with PI serum or no primary antibody (−) (**Cb**). Arrowheads show the position of the native TvLEGU-2 (∼34 and ∼29 kDa) proteins. (**D**) Coomassie Brilliant Blue-stained, purified recombinant TvLEGU-1r protein (CBB; Lane 1). WB assays of TvLEGU-1r incubated with Rα-TvLEGU-1r (1:3000 dilution) (Lane 3), or Rα-TvLEGU-2pep (1:1000 dilution) (Lane 4) antibody or PI serum or only the secondary antibody as a negative control (−) (Lane 2). Arrowhead points to the recombinant protein band TvLEGU-1r (∼46 kDa). (**E**) Coomassie Brilliant Blue-stained, purified recombinant TvLEGU-2r protein (CBB; Lane 1). WB assays of TvLEGU-2r incubated with Rα-TvLEGU-2pep (1:1000 dilution) (Lane 4), or Rα-TvLEGU-1r (1:3000 dilution) (Lane 3) antibody, or PI serum or only the secondary antibody as a negative control (−) (Lane 2). Arrowhead points to the recombinant protein band TvLEGU-2r (∼85 kDa). kDa, molecular weight markers in kilodaltons (Bio-Rad).

To determine the effect of glucose on *tvlegu-2* gene expression, we performed a qRT-PCR assay (Figure 2A) using cDNA from parasites grown in GR or HG conditions and specific primers for the detection of *tvlegu-2* gene mRNA. Figure 2A shows that there was no significant difference in *tvlegu-2* gene mRNA expression between GR and HG conditions (*p* value: 0.7623) in three independent experiments performed in duplicate. The levels of expression were normalized against the *α-tubulin* amplicon for each glucose condition.

### 3.3. Glucose Influences TvLEGU-2 Processing

**Figure 3:**
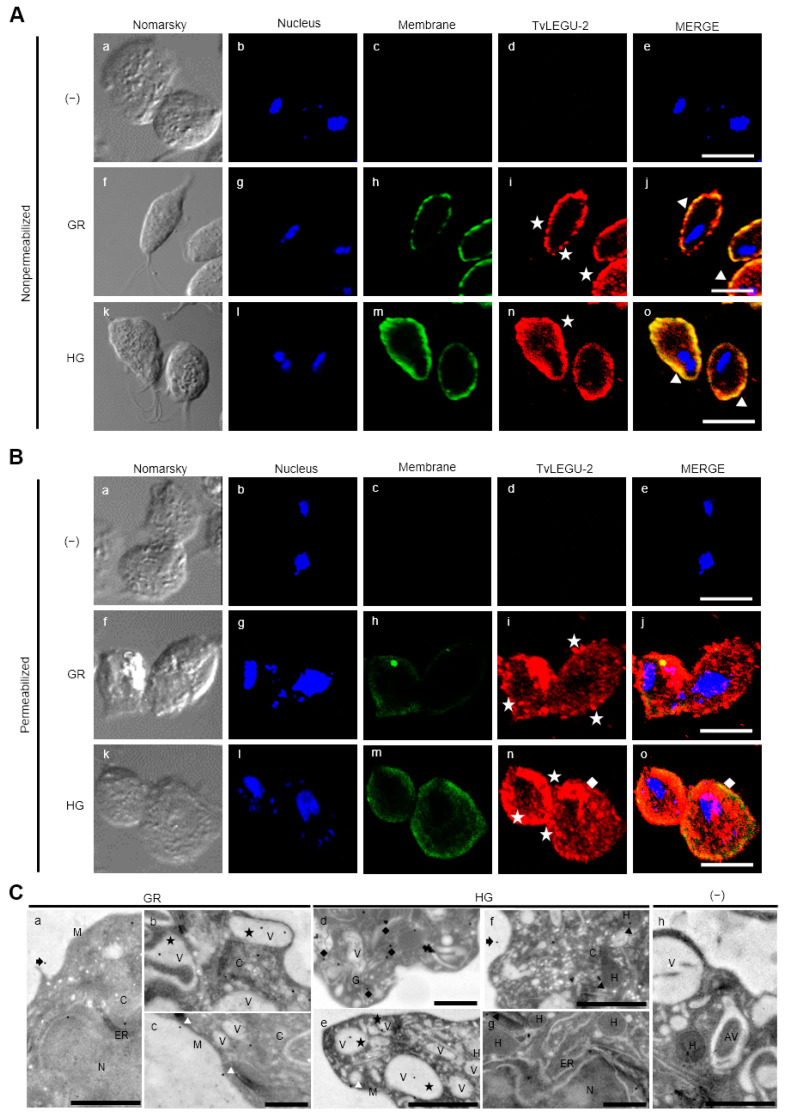
Localization of TvLEGU-2 in *T. vaginalis* under glucose restriction (GR) and high-glucose (HG) conditions. (**A**) IFA of nonpermeabilized parasites under GR (**Af**–**Aj**) and HG (**Ak**–**Ao**) conditions with Mα-TvLEGU-2pep (1:100 dilution) and Rα-Membrane (1:200 dilution) antibodies. Negative control with no primary antibody (−) (**Aa**–**Ae**). TvLEGU-2 (Alexa 647, red), membrane (FITC, green), and nucleus (DAPI, blue). White bar = 10 μm, white arrowheads = membrane colocalization, and white stars = vesicles. (**B**) IFA of permeabilized parasites under GR (**Bf**–**Bj**) and HG (**Bk**–**Bo**) conditions with Mα-TvLEGU-2pep (1:100 dilution) and Rα-Membrane (1:200 dilution) antibodies. The negative control with PI serum or no primary antibody (**Ba**–**Be**). TvLEGU-2 (Alexa 647, red), membrane (FITC, green), and nucleus (DAPI, blue). White bar = 10 μm, white stars = vesicles, white diamond = Golgi apparatus. (**C**) TEM under GR and HG conditions with Mα-TvLEGU-2pep antibody (1:10 dilution) with 30 nm gold particles. Negative control with PI serum or no primary antibody (−). Cytoplasm (C), hydrogenosome (H), membrane (M), nucleus (N), vesicle (V), autophagic vesicle (AV), endoplasmic reticulum (ER), and Golgi apparatus (G). Localization of TvLEGU-2: white arrowheads s = membrane, black arrows = extracellular, black stars = vesicles, black diamonds = Golgi apparatus, and black arrowheads = hydrogenosomes. Scale bar = 500 nm (**Cb**,**Cc**,**Cd**,**Cg**) and scale bar = 1000 nm (**Ca**,**Ce**,**Cf**,**Ch**).

To elucidate the effect of glucose on TvLEGU-2 protein expression in GR- and HG-grown parasites, PREs were prepared, and TvLEGU-2 processing was analyzed by WB assays using a specific antibody generated against a unique TvLEGU-2 peptide (Figure 2B–E). The antibody recognizes protein bands of ∼55, ∼49, ∼34, and ∼29 kDa that correspond to the zymogen (∼49 kDa), precursor with the cleaved C-terminal propeptide (∼34 kDa), and mature peptidase (∼29 kDa). The ∼49 and ∼55 kDa bands could also correspond to PTMs undergone by TvLEGU-2, as in the case of HsLegumain, whose zymogen undergoes glycosylations, phosphorylations, and sumoylation [37] in transit through the endolysosomal system. Although indirect immunofluorescence assays showed no significant difference in the total amount of protein recognized by the antibody (Figure 3B), WB assays indicated that the protein processing and the amount of protein in each band depend on the glucose concentration (Figure 2B, Lanes 10 and 11). In the HG condition, the zymogen with probable PTMs and the precursor with the cleaved C-terminal propeptide are more abundant than in the GR condition, whereas in the GR condition, the ∼29 kDa band of the mature peptidase is more prominent than in the HG condition. These experiments were performed at least three times independently, with identical results. The TvCP2 and TvTIM proteins were used as negative and positive controls for the glucose response, respectively (TvCP2—Lanes 6 and 7; TvTIM—Lanes 8 and 9). An NC membrane incubated with the PI serum or secondary antibody alone was used as a negative control, and no recognition was detected, as expected (Lanes 4 and 5; Supplementary Figure S5A). These data suggest that TvLEGU-2 processing is dependent on glucose concentration.

The C13 family genes in *T. vaginalis* include 10 genes coding for legumain-like peptidases, with varying percentages of identity (24–40%) (Supplementary Figure S1). TvLEGU-2 has a higher identity with TvLEGU-1 (40%) than with the other trichomonad legumains (Supplementary Figure S1B). Thus, we identified a unique peptide of TvLEGU-2 (QSHVMEYGDTSLK) located in an exposed region of the mature peptidase (Supplementary Figure S2A). This peptide is immunogenic (Supplementary Figure S2B), and its sequence does not contain more than three amino acids in a row shared with the other nine legumain proteins (Supplementary Figure S2C). The specificity of the antibodies generated against this unique peptide in TvLEGU-2 was analyzed with a PRE of parasites grown under normal glucose conditions (NG, 25 mM) and subjected to 2-DE and a WB assay. Both rabbit and mouse α-TvLEGU-2pep antibodies recognize two protein spots of ∼34 and ∼29 kDa at the *pI* 4 region that could correspond to the precursor with the cleaved C-terminal propeptide and the mature peptidase, respectively (Figure 2C, d and Supplementary Figure S6A, c). The α-TvLEGU-1r antibody was used as a specificity control because TvLEGU-1 is the peptidase with the highest identity to the unique peptide in TvLEGU-2 (∼50%). Figure 2(Cc) shows the recognition of the *pI* ∼6 region (*pI* ∼6.3, ∼6.5, and ∼6.7) by the α-TvLEGU-1r antibody, with at least 3 spots that show no cross-reaction with the α-TvLEGU-2pep antibody recognition. Furthermore, the α-TvLEGU-2pep antibody did not bind to the TvLEGU-1r protein used as an antigen (Figure 2D), but bound only to the TvLEGU-2r protein used as an antigen (Figure 2E), whereas the α-TvLEGU-1r antibody bound only to the TvLEGU-1r protein used as an antigen (Figure 2D,E). These results show that recognition by the α-TvLEGU-2pep antibody is specific for the TvLEGU-2 peptidase.

### 3.4. Subcellular Localization of TvLEGU-2 in T. vaginalis under Different Glucose Conditions

**Figure 4:**
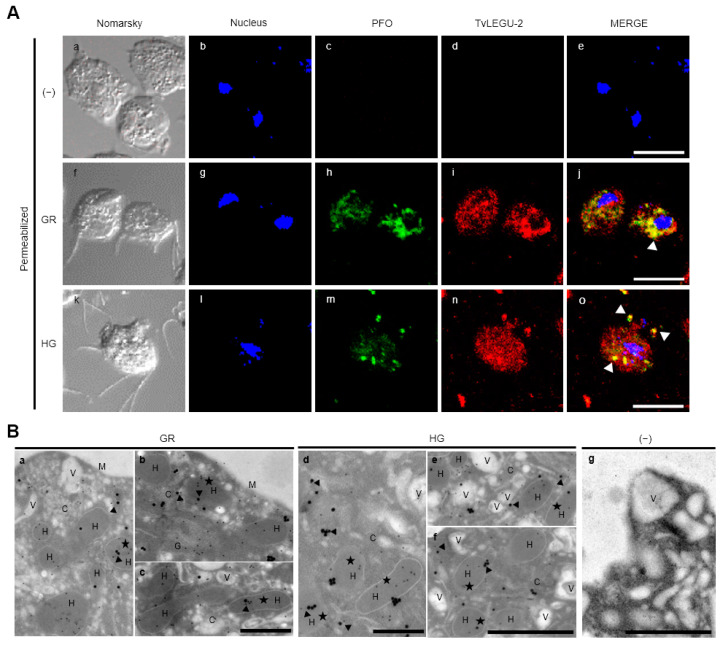
Colocalization of TvLEGU-2 and PFO in *T. vaginalis* hydrogenosomes under glucose restriction (GR) and high-glucose (HG) conditions. (**A**) IFA of permeabilized parasites under GR (**Af**–**Aj**) and HG (**Ak**–**Ao**) conditions with Mα-TvLEGU-2pep (1:100 dilution) and Rα-PFO50 (1:50) antibodies to label hydrogenosomes. Negative control with no primary antibody (−) (**Aa**–**Ae**). TvLEGU-2 (Alexa 647, red), nucleus (DAPI, blue), and PFO (FITC, green). White bar = 10 μm, and the white arrowheads indicate colocalization. (**B**) TEM analysis under GR and HG conditions with Mα-TvLEGU-2pep antibody (1:10 dilution) with 30 nm gold particles and Rα-PFO50 antibody (1:10 dilution) with 15 nm gold particles. Negative control with PI serum or no primary antibody (−). Cytoplasm (C), hydrogenosome (H), membrane (M), and vesicle (V). The black arrowheads indicate the close proximity of both labels, and the black stars indicate the localization of both proteins inside hydrogenosomes. Scale bar = 500 nm (**Ba**–**Bd**), and scale bar = 1000 nm (**Be**–**Bg**).

**Figure 5:**
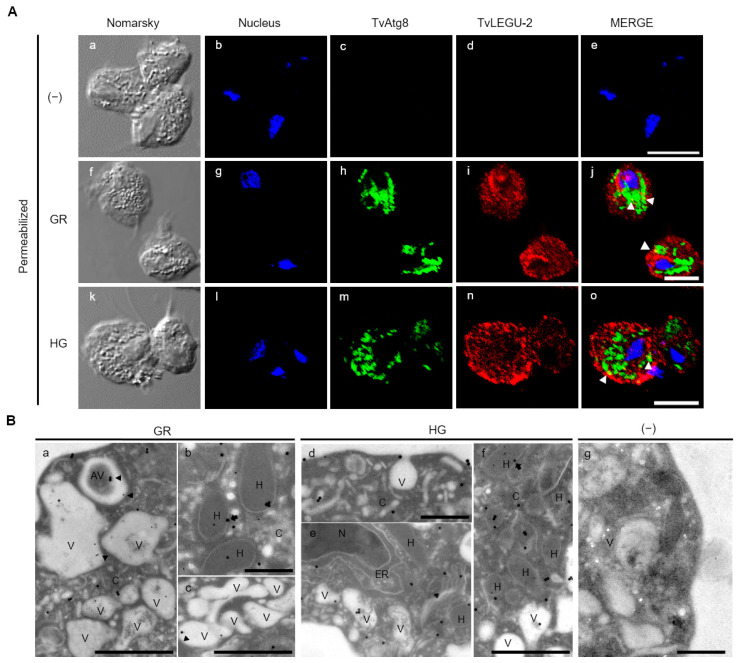
Colocalization of TvLEGU-2 and TvAtg8b in *T. vaginalis* under glucose restriction (GR) and high-glucose (HG) conditions. (**A**) IFA of permeabilized parasites under GR (**Af**–**Aj**) and HG (**Ak**–**Ao**) conditions with Mα-TvLEGU-2pep (1:100) and Rα-rTvATG8b (1:50) antibodies to label autophagosomes. Negative control with PI serum or no primary antibody (−) (**Aa**–**Ae**). TvLEGU-2 (Alexa 647, red), TvAtg8b (FITC, green), and nucleus (DAPI, blue), white bar = 10 μm. The white arrowheads indicate colocalization. (**B**) TEM of parasites grown under GR and HG conditions with Mα-TvLEGU-2pep antibody (1:10 dilution) with 30 nm gold particles and Rα-rTvAtg8b (1:10 dilution) with 15 nm gold particles. Negative control with PI serum or no primary antibody (−). Cytoplasm (C), hydrogenosome (H), nucleus (N), vesicle (V), autophagic vesicle (AV), endoplasmic reticulum (ER), and Golgi apparatus (G). Black arrowhead = TvLEGU-2 and TvAtg8b localization in the same vesicle; black arrow = TvLEGU-2 and TvAtg8b localization in the same hydrogenosome. Scale bar = 500 nm (**Bb**,**Bd**,**Bg**), and scale bar = 1000 nm (**Ba**,**Bc**,**Be**,**Bf**).

To determine the subcellular localization of TvLEGU-2 in parasites grown under different glucose conditions, indirect immunofluorescence assays (IFA) with nonpermeabilized and permeabilized parasites and immunogold labeling combined with transmission electron microscopy (TEM) were performed using the Mα-TvLEGU-2pep antibody (Figure 3, Figure 4 and Figure 5). Figure 3 shows the localization of TvLEGU-2 as a ring on the parasite membrane in nonpermeabilized parasites grown under GR and HG conditions. In both GR and HG conditions, some parasites showed TvLEGU-2 distributed in vesicles around the membrane (Figure 3A, f-j, and k-o, respectively). This localization suggests the active secretion of TvLEGU-2. In permeabilized parasites, TvLEGU-2 was observed in the plasma membrane, cytoplasm, and cytoplasmic vesicles under both glucose conditions (Figure 3(Bi, Bn)). A putative localization in the Golgi apparatus was also observed (Figure 3(Bi, Bn)). To corroborate these localizations, immunogold-TEM assays were performed (Figure 3(Ca–Cg)). Under both glucose conditions, TvLEGU-2 was observed in the parasite membrane, cytoplasm, vesicles, endoplasmic reticulum (ER), hydrogenosomes, extracellular space (Figure 3C), and Golgi apparatus (Figure 3C and Figure 5(Be)). The distribution of TvLEGU-2 was shown to depend on the glucose condition. Under HG conditions TvLEGU-2 was localized in the cytoplasm, hydrogenosomes, and ER (Figure 3(Cd–Cg)). Under GR conditions, the peptidase was localized in the parasite membrane, extracellular space, and vesicles, which was consistent with the observations obtained by IFA, suggesting that TvLEGU-2 could be secreted in the GR conditions (Figure 3(Ca–Cc)). We also tested by IFA the recognition of the Rα-TvLEGU-2pep antibody in permeabilized parasites under both glucose conditions. This antibody also recognized the same subcellular structures (Supplementary Figure S6B). This is not unexpected, since it is known that glucose levels induce a shift in the localization of a wide range of proteins [41,42,43]. In *T. vaginalis*, the availability of glucose affects the localization of other parasite proteins, such as TvCP2, TvAtg8a, TvTIM, and TvCat-D [19,30,44,45]. Thus, differences in the distinct TvLEGU-2 subcellular distribution could be due to the role of glucose in parasite metabolism and alternative functions.

Gold immunolocalization assays combined with TEM suggest that TvLEGU-2 localizes to hydrogenosomes under both HG and GR conditions. To confirm these localization results, IFA and immunogold-TEM colocalization assays using an anti-PFO antibody [33] were performed with pyruvate: ferredoxin oxidoreductase (PFO) protein as a marker for hydrogenosomes (Figure 4). TvLEGU-2 and PFO colocalized in cytoplasmic vesicles under both glucose conditions and, more prominently, in GR conditions (Figure 4A). TEM assays using parasites under both glucose conditions showed that PFO and TvLEGU-2 colocalized in the same hydrogenosomes with a frequency of 28% under GR and 24% under HG conditions (Figure 4B).

Legumains are typically localized in the endolysosomal system, where they are associated with secretion, degradation of components in the autophagic system, and activation of proteases [37]. Thus, to elucidate whether some of the vesicles where TvLEGU-2 was observed in Figure 3 correspond to autophagic vesicles or lysosomes, IFA assays were performed using an anti-TvAtg8b antibody [34] as an autophagic vesicle marker or LysoTracker as a lysosome marker, and TEM immunolocalization assays for TvLEGU-2 and TvAtg8b colocalization were also performed. According to the IFA, TvLEGU-2 and TvAtg8b colocalized poorly in parasites under both glucose conditions (Figure 5A, white arrowheads). According to the TEM assays, the two molecules appeared to colocalize in some vesicles only in the GR condition (14%). Interestingly, the two proteins were observed to colocalize in hydrogenosomes in both GR (7%) and HG (2%) conditions (Figure 5B), which might suggest that TvLEGU-2 plays a degradative role in the autophagosome system, digesting cytoplasmic components, proteins, and organelles such as hydrogenosomes under nutritional stress conditions.

**Figure 6:**
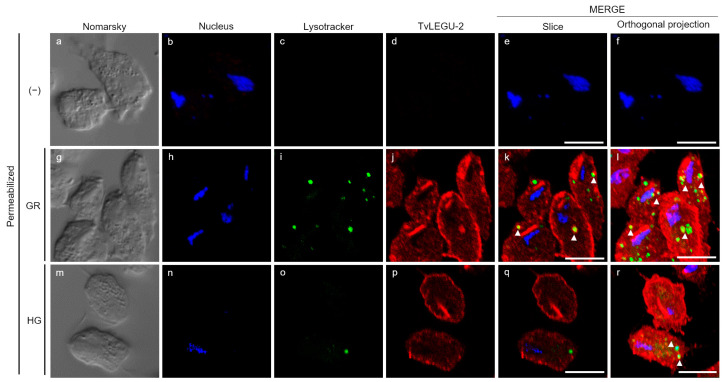
Colocalization of TvLEGU-2 and LysoTracker in *T. vaginalis* under glucose restriction (GR) and high-glucose (HG) conditions. IFA of permeabilized parasites under GR (**g**–**l**) and HG (**m**–**r**) conditions with Mα-TvLEGU-2pep (1:100) and LysoTracker 5 μM to label lysosomes. Negative control with PI serum or no primary antibody (−) (**a**–**f**). TvLEGU-2 (Alexa 647, red), LysoTracker (green), and nucleus (DAPI, blue). White bar = 10 μm. White arrowheads = colocalization of TvLEGU-2 and LysoTracker.

The colocalization assays between TvLEGU-2 and lysosomes showed that in the two glucose conditions, the lysosomes were distributed in different regions of the parasites, as illustrated by the orthogonal projection (Supplementary Figure S3h,m). TvLEGU-2 colocalizes with the LysoTracker marker at some sites in the parasites (Figure 6k,l,q,r), mainly in the GR condition. These results suggest that TvLEGU-2 is found within the endolysosomal system, as has been reported for other legumains [37], as well as appearing elsewhere in *T. vaginalis*, such as the cell membrane, cytoplasm, Golgi complex, secretory vesicles, autophagic vesicles, and hydrogenosomes.

**Figure 7:**
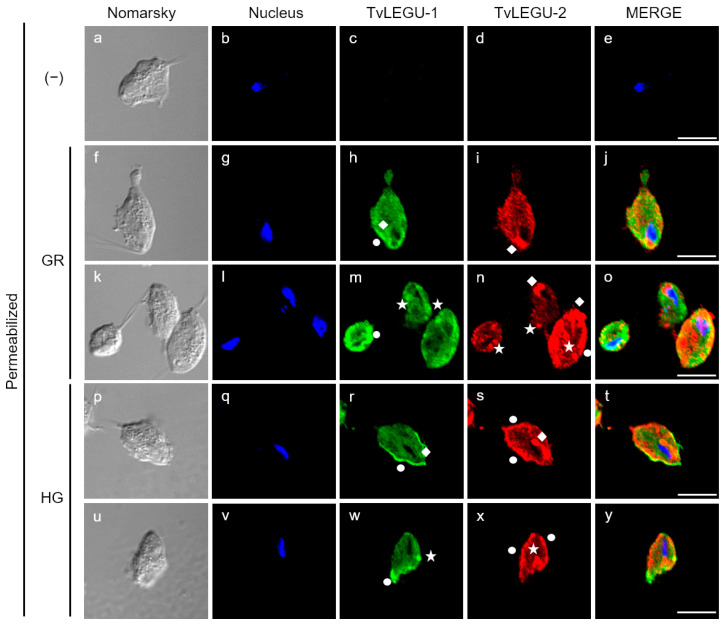
Colocalization of TvLEGU-1 and TvLEGU-2 in *T. vaginalis* under glucose restriction (GR) and high-glucose (HG) conditions. IFA of permeabilized parasites under GR (**f**–**o**) and HG (**p**–**y**) conditions with Mα-TvLEGU-2pep (1:100) and Rα-TvLEGU-1r (1:300) antibodies. Negative control with PI serum or no primary antibody (−) (**a**–**e**). TvLEGU-1 (FITC, green), TvLEGU-2 (Alexa 647, red), and nucleus (DAPI, blue). White bar = 10 μm. White circles = membrane, white diamonds = Golgi apparatus, and white stars = vesicles.

Moreover, by IFA, we examined the colocalization of TvLEGU-1 and TvLEGU-2, the two most abundant legumain proteins in *T. vaginalis*. The colocalization analysis between TvLEGU-1 and TvLEGU-2 showed that TvLEGU-1 is localized in the same organelles as TvLEGU-2, in the membrane, cytoplasm, and Golgi apparatus, but is not always colocalized with TvLEGU-2 (Figure 7), suggesting that both peptidases are synthesized under both glucose conditions and share localization in the same organelles, but are distributed in different spaces throughout the parasite (Figure 7). Interestingly, under GR conditions, there appears to be more colocalization between the two legumain proteins (Figure 7f–o), whereas under HG conditions, the distribution of each legumain appears to be polarized to opposite regions of the parasite membrane (Figure 7p–y), suggesting that the two legumains could have different functions and substrates. The antibodies used against each legumain protein did not cross-react (Figure 2).

### 3.5. TvLEGU-2 Is Secreted under Both Glucose Conditions

**Figure 8:**
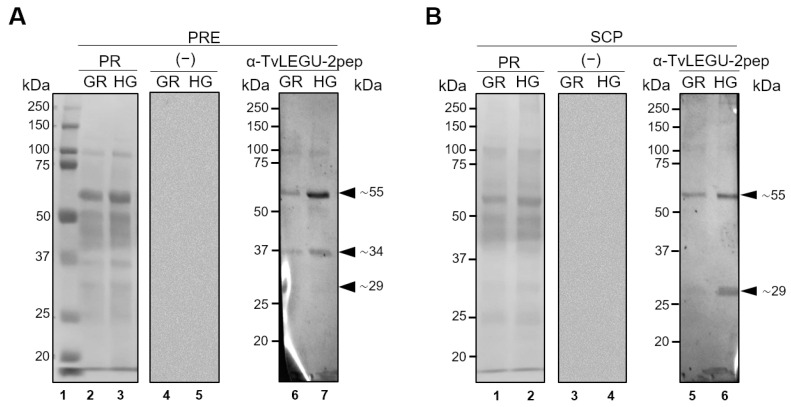
Glucose conditions differentially regulate zymogen and mature peptidase in vitro secretion of TvLEGU-2 by *Trichomonas vaginalis*. (**A**) PREs from parasites grown under GR (<1 mM) and HG (50 mM) conditions were separated on 10% SDS PAGE gels, transferred onto NC membranes, and stained with Ponceau red (PR)- (Lanes 2 and 3: GR and HG, respectively). For WB assays, NC membranes were incubated with different antibodies: Rα-TvLEGU-2pep (1:500 dilution) to detect the TvLEGU-2 protein (Lanes 6 and 7) and PI serum or no primary antibody (−) to serve as a negative control (Lanes 4 and 5). Arrowheads show the position of the native TvLEGU-2 isoform (∼55, ∼34, and ∼29 kDa) proteins. (**B**) Western blot assay of SCPs from parasites grown under GR (<1 mM) and HG (50 mM) conditions that were precipitated by TCA and separated on 10% SDS PAGE gels, transferred onto NC membranes, and PR-stained (Lanes 1 and 2: GR and HG, respectively). For WB assays, NC membranes were incubated with different antibodies: Rα-TvLEGU-2pep (1:500 dilution) to detect the TvLEGU-2 isoforms (Lanes 5 and 6) and PI serum or no primary antibody (−) to serve as a negative control (Lanes 3 and 4). Arrowheads show the positions of the native TvLEGU-2 isoform (∼55 and ∼29 kDa) proteins. kDa, molecular weight markers in kilodaltons (Bio-Rad).

To determine whether TvLEGU-2 is indeed secreted by *T. vaginalis*, an in vitro secretion assay (Figure 8) was performed, and the results were compared with those of a protease-resistant extract (PRE) (Figure 8A). In the PRE, three protein bands of ∼29, ∼34, and ∼55 kDa were detected (Figure 8A). Only the ∼29 and ∼55 kDa protein bands were secreted in both glucose conditions (Figure 8B), and both were more abundant under HG conditions. The ∼29 kDa protein band could correspond to the mature peptidase. These results are similar to the secretion of zymogen and mature legumain peptidase from *H. sapiens* in cancer through the Golgi apparatus direct pathway or the endosomal system indirect pathway [37]. Thus, the HG conditions during the in vitro culture of *T. vaginalis* could induce atypical secretion of these TvLEGU-2 isoforms.

### 3.6. TvLEGU-2 Is an Immunogenic Peptidase Present during T. vaginalis Infection

**Figure 9:**
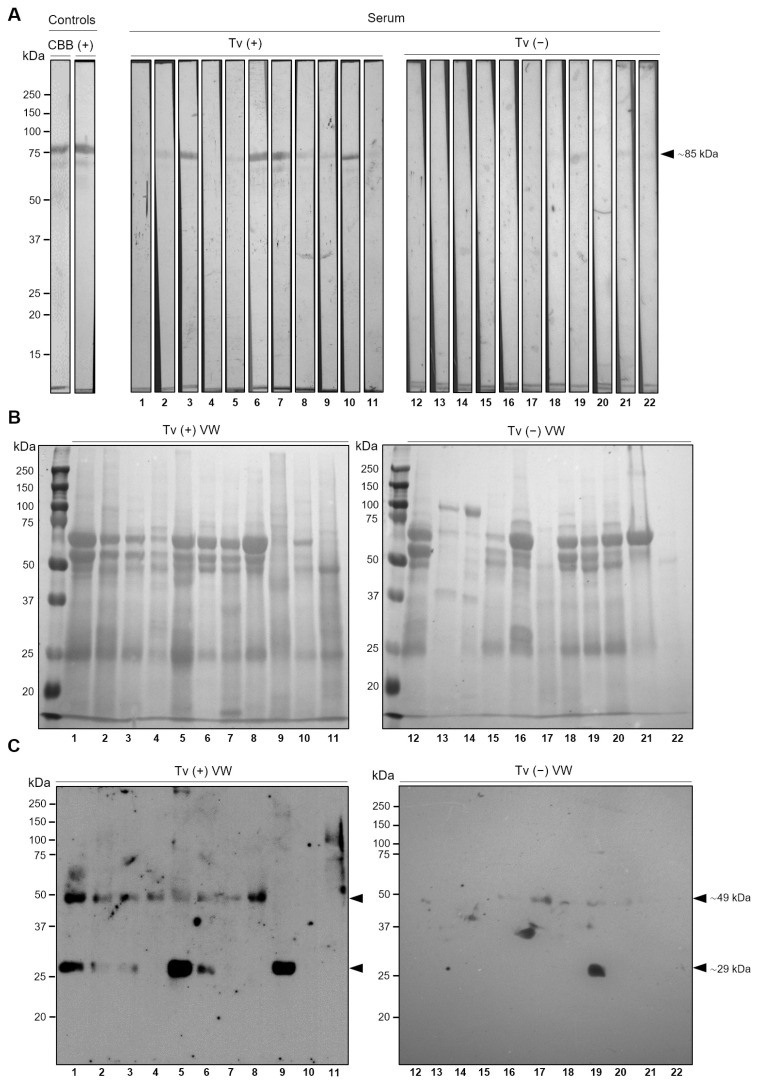
Presence of TvLEGU-2 during trichomonal infection. (**A**) Western blot assay of TvLEGU-2r incubated with Tv (+) and Tv (−) patient sera. Controls of the WB assay using the recombinant TvLEGU-2 Coomassie Brilliant Blue (CBB)-stained 10% SDS PAGE gel for TvLEGU-2r (Lane 1) and recognition by the anti-TvLEGU-2r antibody (1:10,000 dilution; Lane 2). Duplicate gels were transferred onto NC membranes and incubated with different Tv (+) (Lanes 3–12) and Tv (−) (Lanes 13–16) patient sera at 1:25 dilution. Arrowhead points to the recombinant protein band TvLEGU-2r (∼85 kDa). (**B**) Ponceau red (PR)-stained NC membranes showing the patterns of proteins present in 11 VWs from Tv (+) (Lanes 1–11) and 11 VWs from Tv (−−) patients (Lanes 12–22), which were precipitated by TCA, separated by SDS-PAGE using 10% polyacrylamide gels, and transferred onto NC membranes. (**C**) WB assays using the anti-TvLEGU-2r (1:1000 dilution) antibody to incubate the NC membranes described in (**B**). Arrowheads show the positions of the native TvLEGU-2 protein bands (∼49 and ∼29 kDa).

The in vitro secretion of TvLEGU-2 under both glucose conditions (Figure 8B) by *T. vaginalis* suggests that this could also occur during trichomonal infection. Thus, to analyze the expression of TvLEGU-2 during infection, we performed WB assays using TvLEGU-2r as an antigen (Figure 9A) to search for anti-TvLEGU-2 antibodies in sera from patients with vaginitis, 11 Tv (−) and 11 Tv (+), as confirmed by in vitro culture (Table 1). The ∼85 kDa TvLEGU-2r band was detected in the sera from 8/11 Tv (+) patients but in the sera from only 2/11 Tv (−) patients. As a positive control, an anti-TvLEGU-2r antibody was used (Figure 9A). Tv (+) patient sera with no response to TvLEGU-2 could be due to a recent infection, and no antibody against TvLEGU-2 could be detected yet. It could also be that not all Tv (+) patients generate an antibody response to this protein because of individual humoral responses among patients. In contrast, Tv (−) patient sera with TvLEGU-2 antibodies could be due to false-negative cases by in vitro culture, as it has been previously reported [46,47,48], in which this diagnostic method showed 35–70% sensitivity.

The presence of TvLEGU-2 during infection was also analyzed by WB assays of VWs from 11 Tv (+) and 11 Tv (−) patients. The proteins from the VWs of the 22 vaginitis patients (Table 1) were subjected to WB assays using the anti-TvLEGU-2r antibody (Figure 9). Figure 9B shows the Ponceau red-stained protein profile of each VW sample used in this analysis. Figure 9C shows the immunodetection of the ∼29 and ∼49 kDa protein bands of the precursor and mature TvLEGU-2 peptidase in 9/11 Tv (+) VWs (Figure 9C). The anti-TvLEGU-2r antibody detected a ∼49 kDa band in 2/11 and a ∼29 kDa band in 1/11 of the Tv (−) VWs tested (Figure 9C). Additionally, since the recombinant antigen (TvLEGU-2r) used to produce the anti-TvLEGU-2r antibody was produced in *E. coli*, which is a recurrent pathogen in vaginal infections [49], we also analyzed whether there was a cross-reaction of the anti-TvLEGU-2r antibody against any *E. coli* protein by performing WB assays using total protein extracts from *T. vaginalis* and *E. coli* BL21 (DE3). We also used total HeLa cell extracts to confirm that the anti-TvLEGU-2r antibody does not cross-react with human legumains. The anti-TvLEGU-2r antibody recognized only the ∼29 kDa band in *T. vaginalis* extracts, demonstrating that the antibody is specific for the parasite TvLEGU-2 (Supplementary Figure S4). Thus, these data demonstrate that TvLEGU-2 is expressed during infection, is present in vaginal secretions, and is immunogenic, suggesting that it may play a role in *T. vaginalis* pathogenesis in the urogenital tract of trichomoniasis patients.

Indeed, by in silico analysis, we also identified in the TvLEGU-2 aa sequence the molecular fingerprint for hemoglobinase activity by comparing it with the legumains of *Schistosoma mansoni* and *Ixodes ricinus*. The hemoglobinase fingerprint domains are proposed to contribute to hemoglobin degradation either directly or indirectly by the transactivation of other digestive cysteine and aspartic peptidases [50,51]. TvLEGU-2 hemoglobinase domains share 40% identity with both peptidases (Supplementary Figure S7), suggesting that TvLEGU-2 could have hemoglobinase activity and may participate in trichomonal hemolysis. Further analyses should be performed to determine whether TvLEGU-2 is another virulence factor participating in hemolysis as TvCP4 [11].

## 4. Discussion

*Trichomonas vaginalis* has high proteolytic activity compared to that of other parasites [52]. Its degradome comprises ~440 genes encoding peptidases [8], mainly those of the CP type. The CPs of *T. vaginalis* are virulence factors involved in the pathogenesis of the parasite [12]. Some are highly immunogenic and are found in the secretions of infected patients [9,14]. In addition, as *T. vaginalis* is an extracellular parasite, it has adopted strategies allowing it to differentially regulate the expression of its virulence factors depending on several environmental conditions in the host, such as the vaginal pH, temperature, and levels of iron, glucose, and polyamines [16,19,44]. During trichomoniasis, the glucose levels in the vagina fluctuate [30], and these changes in the vaginal environment differentially modulate trichomonal cytotoxicity and apoptosis induction in host cells [19]. These effects are related to CPs of 65 kDa (TvCP65) [17] and 39 kDa (TvCP39) [18,53,54], and some CPs of the 30 kDa region belonging to clan CA, such as TvCP2 [19,20] and TvCP3, TvCP4, and TvCP12 [55,56] (our unpublished data). However, the mechanisms by which individual cells respond to changes in the intracellular levels of glucose are not fully understood.

In this study, we showed that glucose influences TvLEGU-2 peptidase localization, secretion, and processing and that TvLEGU-2 is expressed during infection and is an immunogenic protein. The in silico analysis of TvLEGU-2 showed that it contains several functional domains: SP, protease, AP, and LSAM domains, which are also present in human legumain [37], although the N-terminal propeptide in human legumain was not found in the TvLEGU-2 sequence. In addition, TvLEGU-2 showed a tumor necrosis factor receptor-associated factor 6 (TRAF6) protein binding motif (XXPXEXXAr/Ac) that is, in human legumain, reported to promote protease stability at pH values near 7.0 [57]. Computational analysis suggested the presence of several PTMs, such as phosphorylation, O-glycosylation, and acetylation (Figure 1(Ba)). In other proteins, PTMs, such as phosphorylation and acetylation, are related to the regulation of function, stability, cellular localization, and protein–protein interactions [58]. Glycosylation plays an important role in the localization, internalization, transport, secretion, and function of human legumain [59]. In *T. vaginalis*, TvLEGU-1 is a multiphosphorylated peptidase with tyrosine and threonine phosphorylations. It also contains O- and N-glycosylation (Rendón-Gandarilla unpublished data). Further analysis needs to be performed to determine whether TvLEGU-2 contains some of the predicted PTMs; however, its presence in the Golgi complex together with higher molecular weight protein bands (∼55 and ∼49 kDa bands) could suggest possible glycosylation.

As in the case of human legumain, in silico predictions suggested that TvLEGU-2 undergoes several processing steps: zymogen, precursor with the cleaved SP, C-terminal propeptide, and mature peptidase. WB assays of PRE under GR and HG conditions showed several bands of ∼55, ∼49, ∼34, and ∼29 kDa (Figure 2B). Some of these bands could correspond to the zymogen (∼45 kDa), precursor with the cleaved C-terminal propeptide (∼34 kDa), and mature peptidase (∼29 kDa), and the ∼55 kDa bands (Figure 1(Bc)) could correspond to a glycosylated isoform, as in TvLEGU-1 (Rendón-Gandarilla unpublished data). Interestingly, glucose was found to affect the processing of TvLEGU-2, with a more precursor obtained in HG and a more mature peptidase in GR (Figure 2B). In humans, legumain processing occurs in lysosomes and is dependent on pH. A report by Moheimani et al. [60] demonstrated that in two macrophage lines (HMDM and J774A.1), high concentrations of glucose affect lysosomal function, their number, the accumulation of modified proteins, and reduce the proteolytic activity of cathepsins B, L, D, and S. Cathepsins B and L are activated by legumains [37]. Hence, dysregulation of lysosomal activity would affect the processing of legumain and, thus, the activity of lysosomal proteases. In *T. vaginalis* cultured under HG conditions, we observed reduced lysosomal signal (Figure 6) as well as less cathepsin L activity as compared with GR conditions [19]. Therefore, we hypothesize that HG conditions also produce dysregulation of the lysosomal activity in *T. vaginalis* that could affect the processing of TvLEGU-2. The fluctuating concentrations of glucose in the vaginal microenvironment during trichomoniasis [30] suggest that *T. vaginalis* could form different processing states of TvLEGU-2, thus varying its activity and localization in the parasite.

TvLEGU-2 is localized in multiple parasite compartments, particularly the Golgi apparatus, ER, vesicles, and cell membrane (Figure 3A), but we can also find it in the flagellum (Figure 4(Ao)) and axostyle (Figure 6r). These results suggest that TvLEGU-2 partially shares the typical localization of legumains, including its passage through the ER and translocation to the Golgi apparatus, where it is either directed to the endolysosomal system for activation and function as a peptidase or secreted via endolysosomes or by secretory vesicles via the Golgi apparatus [37]. Interestingly, TvLEGU-2 was also found to colocalize in hydrogenosomes with the hydrogenosomal protein PFO (Figure 4). Its function in this organelle is not yet clear, but TvLEGU-2 also colocalize in some hydrogenosomes with the Atg8 protein, a key protein in autophagy [61]. This result suggests the participation of TvLEGU-2 in the degradation of hydrogenosomes by the autophagic system, that is, hydrogenophagy [33,34,62]. Our results also show that some of the vesicles containing TvLEGU-2 (Figure 3C) are lysosomes (Figure 6). This localization is consistent with previous trichomonad lysosome mass spectrometry data [27] and with the results reported for mammalian legumains [37]. Moreover, this multiple localization of TvLEGU-2 is similar to that of TvLEGU-1, another *T. vaginalis* legumain, which was also found in the cytoplasm, cell membrane, Golgi apparatus, hydrogenosomes, and lysosomes [22]. Interestingly, our colocalization assays showed that although TvLEGU-1 and TvLEGU-2 are the two most abundant legumains in the parasite [10,24,25,26,27,28,29,63,64], they are localized in different compartments at the same time in *T. vaginalis* (Figure 7), suggesting that they could play different roles in *T. vaginalis* under certain culture conditions.

In addition to their main localization in the endolysosomal system, legumains are secreted into the extracellular milieu and are suggested to perform multiple extracellular functions [65]. Thus, in this study, we explored whether glucose affected TvLEGU-2 in vitro secretion (Figure 8). Interestingly, we observed that both the zymogen and the mature peptidase were secreted. Glucose increased the secretion of mature TvLEGU-2. Secretion in several cell types and organisms is induced in the presence of glucose [66,67,68]. Moreover, in bacteria of the genera *Enterococcus*, *Pseudomonas aeruginosa*, *Escherichia coli*, and *Staphylococcus aureus*, cultures in the presence of glucose promote the secretion of ATP [68]. These results are consistent with previous reports on human legumain. The secretion of both the zymogen and the mature peptidase of human legumain has been reported [57,69,70,71]. A relationship has been found between the secretion of human legumain zymogen and the progression of metastasis in human breast cancer [57], and the secretion of the mature form has been linked to carotid atherosclerosis [69]. The functions of zymogen and mature TvLEGU-2 peptidase in the pathogenesis of *T. vaginalis* are not yet known and require further study.

Furthermore, TvLEGU-1 is another peptidase that is secreted in vitro [22], and together with TvLEGU-2, it has been identified in secretory proteomes [24,25,26]. In contrast to TvLEGU-2, however, only the TvLEGU-1 mature peptidase has been detected. This could be relevant to controlling the activity of TvLEGU-2.

The in vitro secretion results of TvLEGU-2 led us to analyze the expression and presence of TvLEGU-2 during trichomoniasis. Our results demonstrated that TvLEGU-2 protein is expressed and is present during infection, as the peptidase was detected in the secretions of Tv (+) patients (Figure 9C) and is recognized by the immune systems of patients infected with *T. vaginalis*: anti-TvLEGU-2 antibodies were detected in the sera of Tv (+) patients (Figure 9A). Interestingly, the ∼29 kDa band was detected in both VWs and in vitro parasite secretion products (Figure 8B and Figure 9B), suggesting that this band corresponds to the mature peptidase. In contrast, the ∼55 kDa band was detected only in the parasite in vitro secretion, whereas the ∼49 kDa band was detected in the VWs. This could be due to differences in the microenvironment between the two samples, as the parasite in contact with vaginal cells employs a different level of posttranslational modifications. These data are consistent with the results of *T. vaginalis* immunoproteome analysis, in which TvLEGU-2 was also detected by antibodies anti-TvLEGU-2 present in the sera from Tv (+) patients [10], suggesting that TvLEGU-2 has a potential role in trichomoniasis and could serve as a biomarker for the disease. These results are consistent with those of other legumains from *Opisthorchis viverrini*, *Clonorchis sinensis*, and *Angiostrongylus cantonensis*, whose legumains have been proposed as biomarkers of disease [72,73,74,75], and in *Dictyocaulus viviparus*, whose legumain is being tested as a target for a vaccine against this helminth [76].

## 5. Conclusions

In conclusion, in this study, we determined that glucose affects the processing, localization, and secretion of TvLEGU-2. This peptidase was found in vaginal secretions, and antibodies against TvLEGU-2 were detected in the sera of patients with trichomoniasis, supporting its potential as a virulence factor and as a biomarker for the disease.

## 6. Patents

This study will be submitted to IMPI for patent registration.

## Figures and Tables

**Table 1:** Characteristics of biological samples from patients with vaginitis used in Western blot assays shown in Figure 9.

		VW Microscopic Analysis ^b^						
Sample Number ^a^	Patient ID	VEC ^d^	WBC ^e^	Bacteria	Yeast	Tv ^f^	Tv Culture	Serum ^c^
1	HGMM 718	+	+	+++	−	+	+	−
2	HGMM 766	+	+	+++	−	+	+	+
3	HGMM 826	++	++	+++	−	+	+	+
4	HGMM 827	++	+	+++	−	+	+	−
5	HGMM 842	++	+	+++	−	+	+	+
6	HGMM 949	+	+	+++	−	+	+	+
7	HGMM 990	++	+++	+++	+	+	+	+
8	HGMM 999	++	++	++	+	+	+	+
9	HGMM 1000	++	++	++	−	+	+	+
10	HGMM 1005	+++	++	++	−	+	+	+
11	HGMM 1041	+	++	++	−	+	+	−
12	HGMM 983	+	+	++	−	−	−	−
13	HGMM 984	++	++	++	−	−	−	−
14	HGMM 985	++	++	++	−	−	−	−
15	HGMM 997	++	+++	+++	+	−	−	−
16	HGMM 1012	+++	++	+++	−	−	−	−
17	HGMM 1021	+++	++	+++	+	−	−	−
18	HGMM 1025	+++	++	++	−	−	−	−
19	HGMM 1027	++	++	++	−	−	−	+
20	HGMM 1031	+++	+++	++	+	−	−	−
21	HGMM 1034	+	+	+	+	−	−	+
22	HGMM 1035	++	++	++	−	−	−	−

Samples of vaginal washes (VWs) and sera were obtained from enrolled patients attending for an annual pap smear examination at the “Medicina Preventiva del Hospital General de México” (HGMM). Patients accepted to participate after signing the consent form approved in Protocol No. DI/14/204/03/010 by the HGM ethical committee. The presence of Tv detected in the microscopic analysis was confirmed by in vitro culture of vaginal secretions (Tv culture). All patients were diagnosed with cervicovaginitis. ^a^ The patient sample numbers from 1 to 11 of WVs and sera are from Tv (+) patients, and those from 12 to 22 are from Tv (−) patients. These samples were used in the WBs shown in Figure 9. ^b^ Patient WVs were TCA-precipitated for the WB assay presented in Figure 9C using anti-TvLEGU-2r serum as the primary antibody. The protein profile of each VW was shown in Figure 9B. Signs (+ or −) show the abundance of cellular components as follows: +, few; ++, moderate; +++ abundant; and − absent. ^c^ Patient serum at 1:25 dilution for the WB assay presented in Figure 9A using TvLEGU-2r as an antigen. ^d^ VEC, presence of vaginal epithelial cells. ^e^ WBC, presence of white blood cells. ^f^ Tv, presence of moving parasites confirmed by in vitro culture of VW.

## Data Availability

The data presented in this study are available on request from the corresponding author.

## Supplementary Materials

The following supporting information can be downloaded at: https://www.mdpi.com/article/10.3390/pathogens13020119/s1, Figure S1: Multiple alignments of *T. vaginalis* legumains. Figure S2: Characteristics of the synthetic peptide QSHVMEYGDTSLK. Figure S3: Orthogonal projection of the colocalization of TvLEGU-2 and LysoTracker in *T. vaginalis* under glucose restriction (GR) and high-glucose (HG) conditions. Figure S4: The anti-TvLEGU-2r antibody is specific. Figure S5: The absence of recognition of Tv proteins by preimmune serum from individual mouse and rabbit used to produce the mouse and rabbit anti-TvLEGU-2pep antibodies. Figure S6: A. Specificity of the mouse anti-TvLEGU-2pep antibody. B. Comparative recognition of subcellular structures by the mouse and rabbit anti-TvLEGU-2pep antibodies by IFA. Figure S7. TvLEGU-2 may have hemoglobinase activity.
