# Supplementary material for: Trichomonas vaginalis Legumain-2, TvLEGU-2, Is an Immunogenic Cysteine Peptidase Expressed during Trichomonal Infection
Source: Pathogens. 2024 Jan 27;13(2):119. doi: 10.3390/pathogens13020119 (PMC10892250; doi:10.3390/pathogens13020119)
Supplement: Supplementary file 1 [file pathogens-13-00119-s001.zip › New Supplementary Figure S5 260124.pdf]

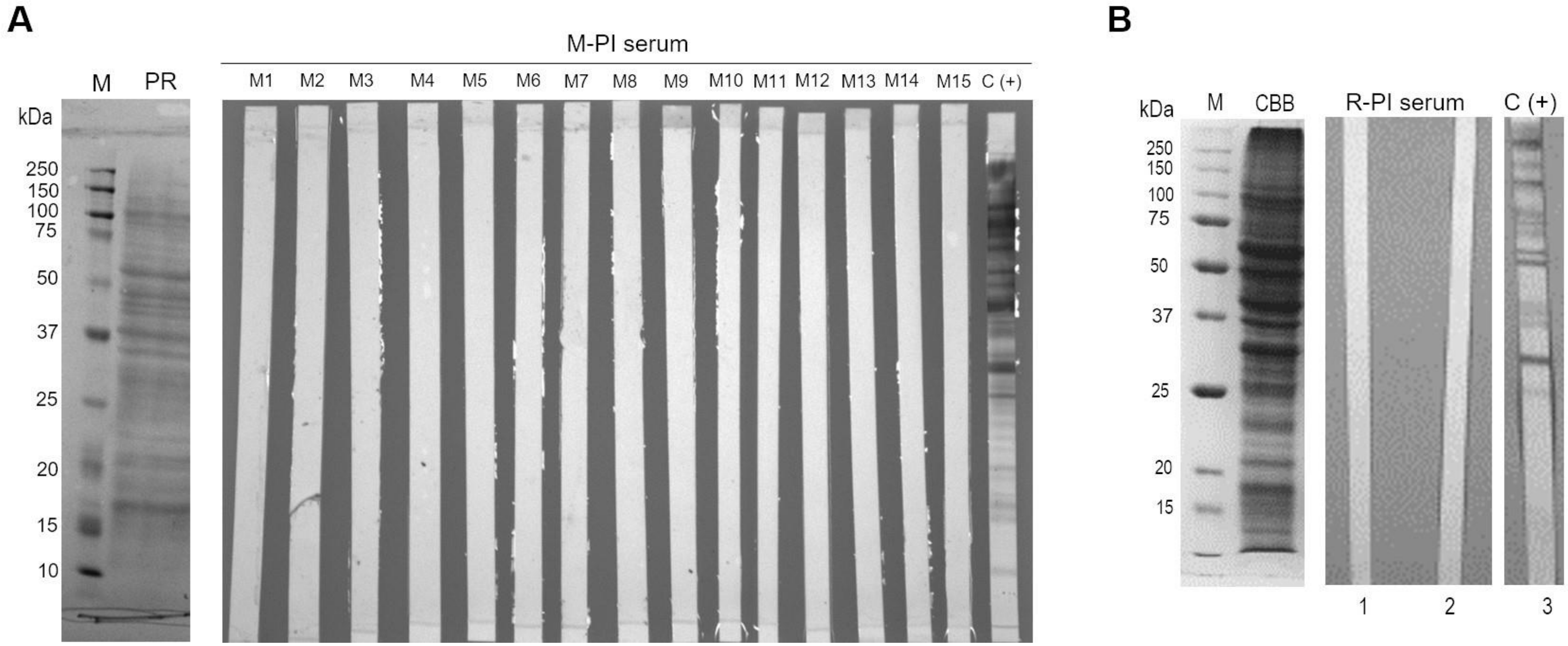

Supplementary Figure S5. Absence of recognition of Tv proteins by preimmune serum from individual mouse and rabbit used to produce the mouse and rabbit anti-TvLEGU-2pep antibodies. A. Western blot assay of total protein extract (TPE) from parasites of the CNCD188 *T. vaginalis* (Tv) isolate. Ponceau Red (PR)-stained 12% SDS PAGE gel for TPE protein pattern. For WB assays, NC membranes containing Tv TPE were incubated with 15 different mouse preimmune (PI) sera (1:1000 dilution) to follow reactivity against Tv extract. A rabbit α-Tv (1:1000 dilution) antibody was used as a positive control [C (+)]. M, molecular weight markers in kilodaltons (kDa). **B**. Preimmune (PI) serum of a rabbit used to produce the R α-TvLEGU-2pep antibody. Western blot assay using Tv TPEs from the CNCD188 *T. vaginalis* isolate. Coomassie brilliant blue (CBB)-stained 12% SDS PAGE gel for TPE protein pattern. For WB assays, lanes 1 and 2, NC membranes containing Tv TPE incubated with a rabbit preimmune (R-PI) serum at 1:100 and 1:1000 dilutions, respectively to follow reactivity against Tv extract. A rabbit α-Tv (1:1000 dilution) antibody was used as a positive control [C (+)].
