# Supplementary material for: Trichomonas vaginalis Legumain-2, TvLEGU-2, Is an Immunogenic Cysteine Peptidase Expressed during Trichomonal Infection
Source: Pathogens. 2024 Jan 27;13(2):119. doi: 10.3390/pathogens13020119 (PMC10892250; doi:10.3390/pathogens13020119)
Supplement: Supplementary file 1 [file pathogens-13-00119-s001.zip › New Supplementary Figure S6 260124.pdf]

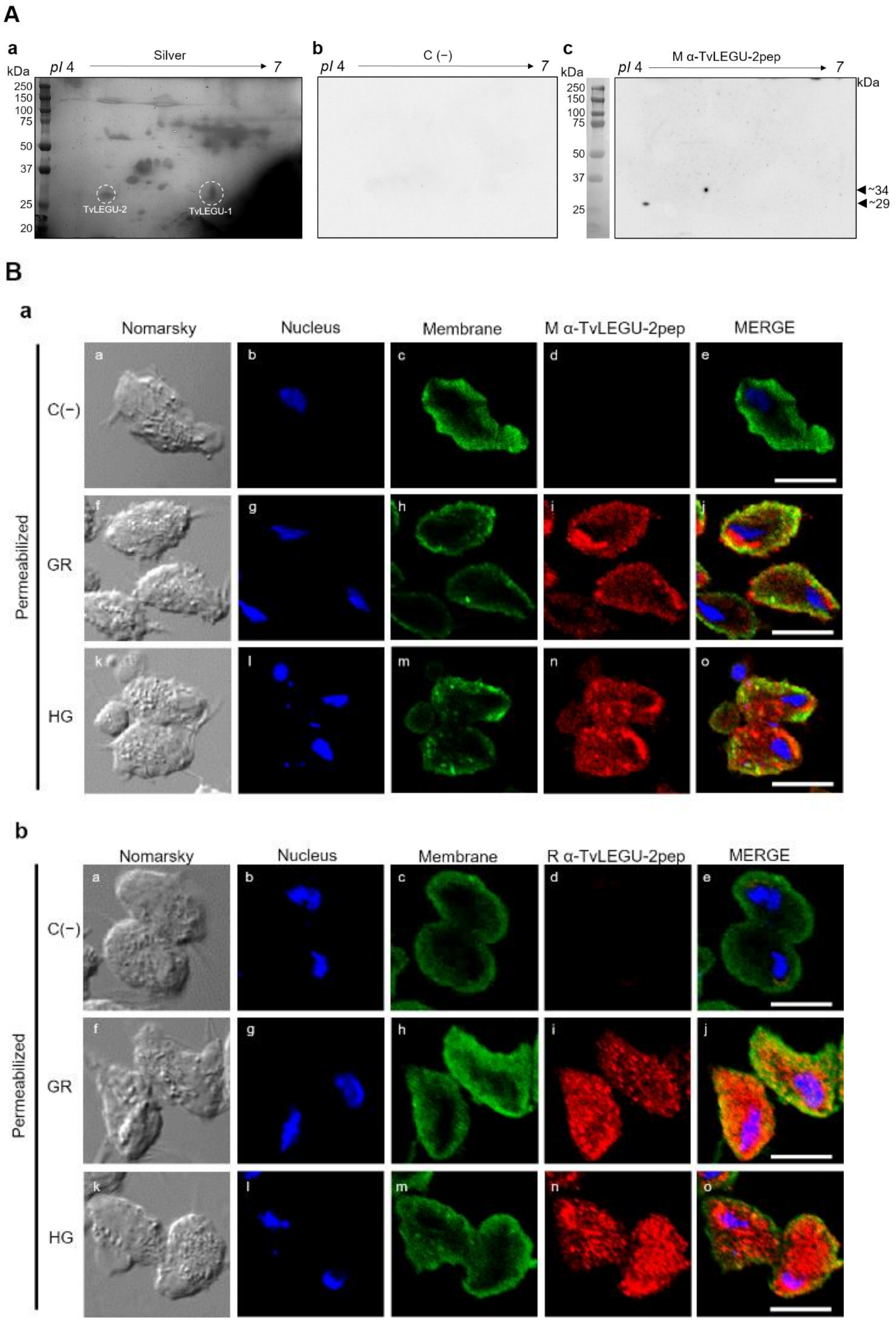

A
a
Silver
pI 4
7
kDa
250
150
100
75
50
37
25
20
TvLEGU-2
TvLEGU-1
b
C (−)
pI 4
7
c
M α-TvLEGU-2pep
kDa
250
150
100
75
50
37
25
~34
~29
B
a
Nomarsky
Nucleus
Membrane
M α-TvLEGU-2pep
MERGE
Permeabilized
C(−)
GR
HG
b
Nomarsky
Nucleus
Membrane
R α-TvLEGU-2pep
MERGE
Permeabilized
C(−)
GR
HG

**Supplementary Figure S6. A. Specificity of the mouse anti-TvLEGU-2pep antibody. B. Comparative recognition of subcellular structures by the mouse and rabbit anti-TvLEGU-2pep antibodies by IFA. A.** Silver-stained 2-DE protease-resistant extracts (PREs) from parasites grown under normal glucose conditions (25 mM) (panel a). WB of duplicate gels transferred onto NC membranes incubated with the M α-TvLEGU-2pep antibody (1:100 dilution) (panel c) to follow the TvLEGU-2 protein in PREs, or with the PI serum as a negative control (panel b). Arrowheads show the position of the native TvLEGU-2 (~34 and ~29 kDa) proteins. kDa, molecular weight markers in kilodaltons (Bio-Rad). Dashed white circles indicate the position of TvLEGU-2 and TvLEGU-1 protein spots in the silver-stained gel image. **B.** Indirect immunofluorescence assays of fixed and permeabilized parasites grown under GR (f-j) and HG (k-o) conditions incubated with (**a**) M α-TvLEGU-2pep (1:100 dilution) and R α-CNCD188 extract as a membrane marker (1:200 dilution) antibodies. (**b**) R α-TvLEGU-2pep (1:200 dilution) and M α-CNCD188 extract as membrane marker (1:100 dilution) antibodies. Negative control, C (−) with the PI serum or without anti-TvLEGU-2pep primary antibody (a-e). TvLEGU-2 (Alexa 647, red), membrane (FITC, green), and nucleus (DAPI, blue), white bar, 10 µm.
