# Supplementary material for: Trichomonas vaginalis Legumain-2, TvLEGU-2, Is an Immunogenic Cysteine Peptidase Expressed during Trichomonal Infection
Source: Pathogens. 2024 Jan 27;13(2):119. doi: 10.3390/pathogens13020119 (PMC10892250; doi:10.3390/pathogens13020119)
Supplement: Supplementary file 1 [file pathogens-13-00119-s001.zip › New Supplementary Figure S7 260124.pdf]

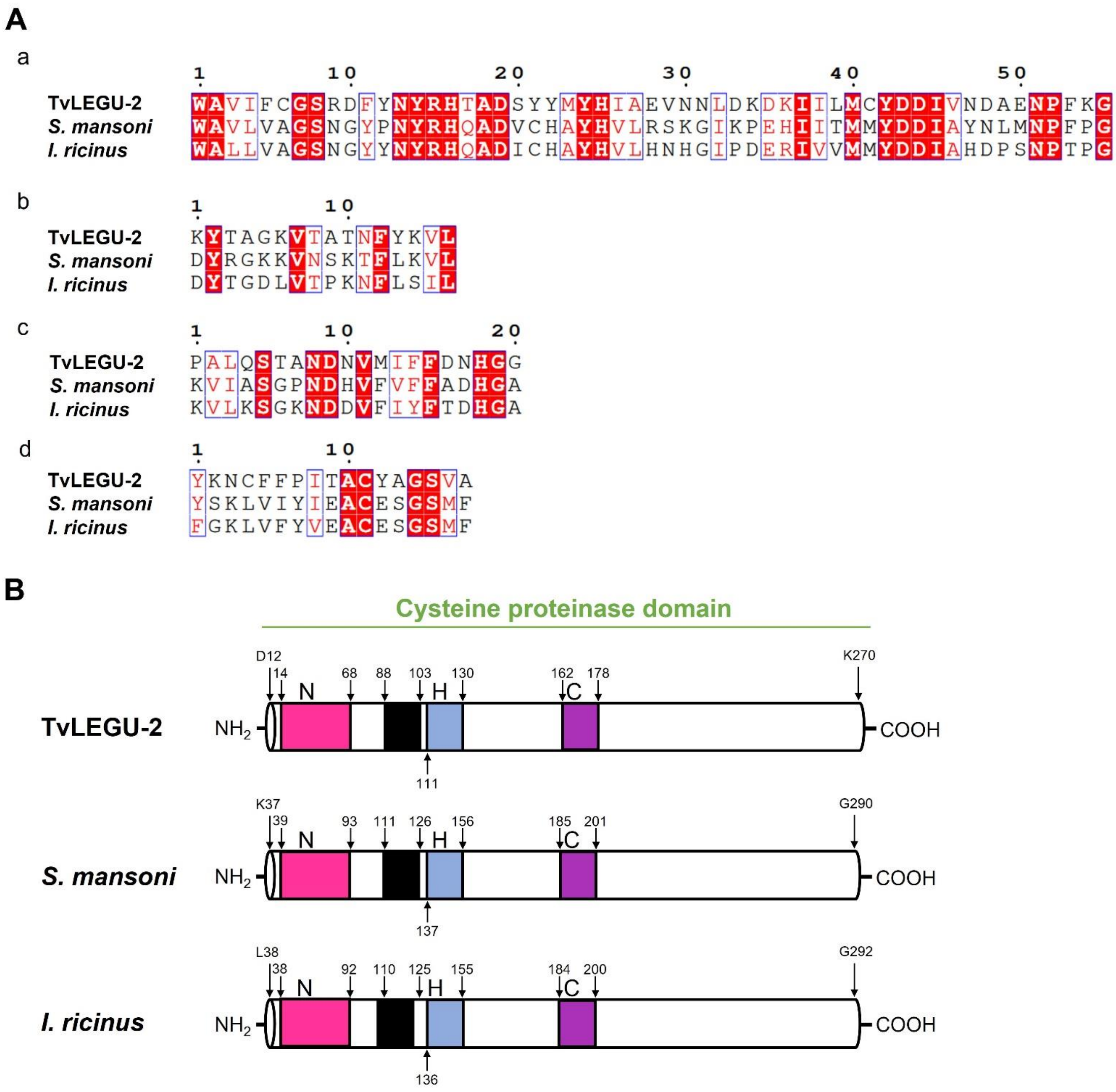

Supplementary Figure S7. TvLEGU-2 may have hemoglobinase activity. A. Multiple alignment of hemoglobinase domain amino acid sequences of *Trichomonas vaginalis* TvLEGU-2 (TVAG_385340), *Schistosoma mansoni* (GenBank AJ250582) and *Ixodes ricinus* (GenBank AY584752) in the legumain catalytic región of the five domains 1, 2, 3, 4 and 5, respectively (a, b, c, and d). Shadowed aa, identical aa residues; red letter, conserved aa residues; black letter, distinct aa residues. **B.** Schematic representation of the multiple alignment among the three legumain catalytic regions shown in **A**. Domains 1 and 2 (pink box), domain 3 (black box), domain 4 (blue box), domain 5 (purple box). Black arrows, point to the aa residue position of each domain.
