# Supplementary material for: Trichomonas vaginalis Legumain-2, TvLEGU-2, Is an Immunogenic Cysteine Peptidase Expressed during Trichomonal Infection
Source: Pathogens. 2024 Jan 27;13(2):119. doi: 10.3390/pathogens13020119 (PMC10892250; doi:10.3390/pathogens13020119)
Supplement: Supplementary file 1 [file pathogens-13-00119-s001.zip › Supplementary Figure S1 sent 260124.pdf]

A

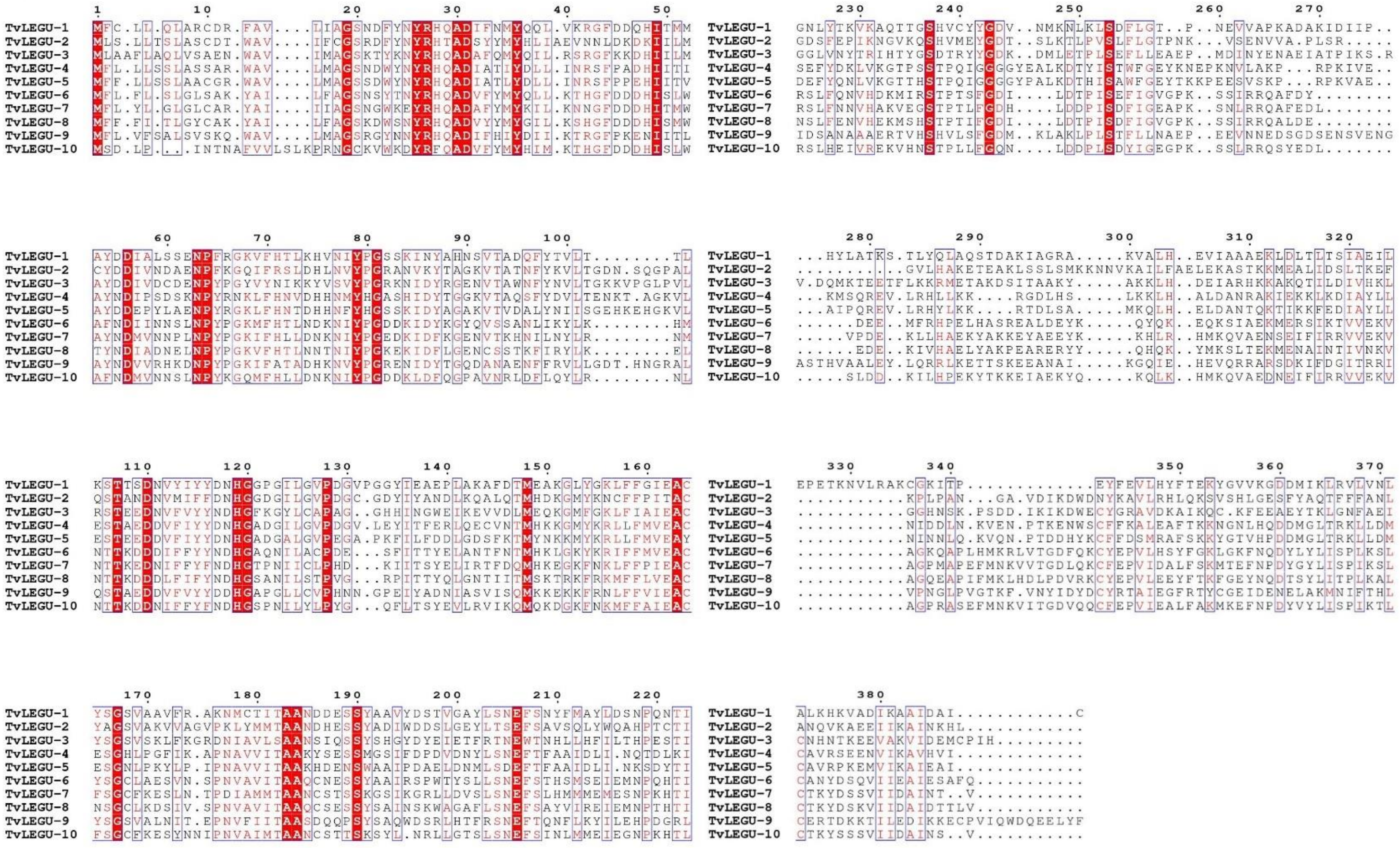

B

	% of identity
	TvLEGU-2
TvLEGU-1	40
TvLEGU-2	100
TvLEGU-3	30
TvLEGU-4	33
TvLEGU-5	31
TvLEGU-6	28
TvLEGU-7	28
TvLEGU-8	27
TvLEGU-9	32
TvLEGU-10	24

Supplementary Figure S1. Multiple alignment of *T. vaginalis* legumains. A. Multiple alignment was performed using the amino acid sequences of legumains from *Trichomonas vaginalis:* TvLEGU-1 (TVAG_426660), TvLEGU-2 (TVAG_385340), TvLEGU-3 (TVAG_050390), TvLEGU-4 (TVAG_328450), TvLEGU-5 (TVAG_060430), TvLEGU-6 (TVAG_185540), TvLEGU-7 (TVAG_035520), TvLEGU-8 (TVAG_068410), TvLEGU-9 (TVAG_305110), and TvLEGU-10 (TVAG_277470). The T-Coffee server (https://tcoffee.crg.eu/apps/tcoffee/index.html) was used for identity analysis with modification using the ESPript 3.0 server (https://espript.ibcp.fr/ESPript/ESPript/index.php). Amino acids marked in red are conserved in all proteins, and amino acids in blue boxes are conserved in almost all proteins. Amino acid lettering in red indicates conserved amino acids and black lettering indicates no conserved amino acids. **B.** Table of *T. vaginalis* legumain identity percentages compared to TvLEGU-2.
