# Supplementary material for: Trichomonas vaginalis Legumain-2, TvLEGU-2, Is an Immunogenic Cysteine Peptidase Expressed during Trichomonal Infection
Source: Pathogens. 2024 Jan 27;13(2):119. doi: 10.3390/pathogens13020119 (PMC10892250; doi:10.3390/pathogens13020119)
Supplement: Supplementary file 1 [file pathogens-13-00119-s001.zip › Supplementary Figure S2 sent 260124.pdf]

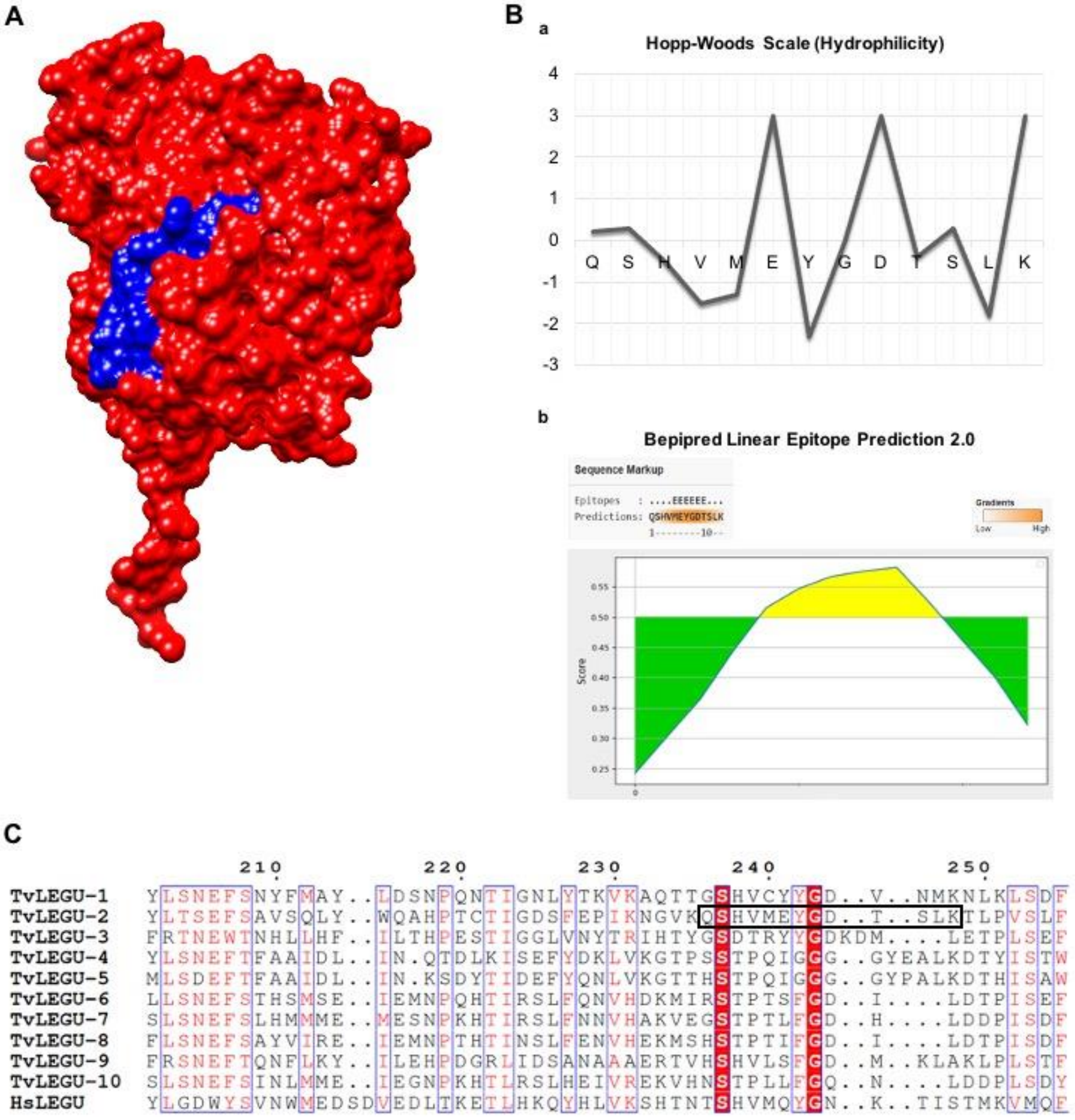

Supplementary Figure S2. Characteristics of the synthetic peptide QSHVMEYGDTSLK. A. Localization of the QSHVMEYGDTSLK peptide in TvLEGU-2. Blue: QSHVMEYGDTSLK peptide localized in an exposed area of the protein. **B.** Analysis of peptide immunogenicity. **a.** Hopp-Woods scale of amino acid hydrophilicity. **b.** Prediction of epitopes recognized by B lymphocytes by the BepiPred-2.0 server. **C.** Multiple peptide region alignment was performed using the amino acid sequences of legumains from the following organisms: *Trichomonas vaginalis:* TvLEGU-1 (TVAG_426660), TvLEGU-2 (TVAG_385340), TvLEGU-3 (TVAG_050390), TvLEGU-4 (TVAG_328450), TvLEGU-5 (TVAG_060430), TvLEGU-6 (TVAG_185540), TvLEGU-7 (TVAG_035520), TvLEGU-8 (TVAG_068410), TvLEGU-9 (TVAG_305110), and TvLEGU-10 (TVAG_277470) and *Homo sapiens* (GenBank Y09862). The T-Cofee server (https://tcoffee.crg.eu/apps/tcoffee/index.html) was used for identity analysis with modification in the ESPript 3.0 server (https://espript.ibcp.fr/ESPript/ESPript/index.php) and the AlphaFold Protein Structure Database (https://AlphaFold.ebi.ac.uk/) to obtain the three-dimensional (3D) model of the native TvLEGU-2 structure. The models were visualized with UCSF Chimera 1.16. The black box indicates the peptide.
