# Supplementary material for: Trichomonas vaginalis Legumain-2, TvLEGU-2, Is an Immunogenic Cysteine Peptidase Expressed during Trichomonal Infection
Source: Pathogens. 2024 Jan 27;13(2):119. doi: 10.3390/pathogens13020119 (PMC10892250; doi:10.3390/pathogens13020119)
Supplement: Supplementary file 1 [file pathogens-13-00119-s001.zip › Supplementary Figure S3 sent 260124.pdf]

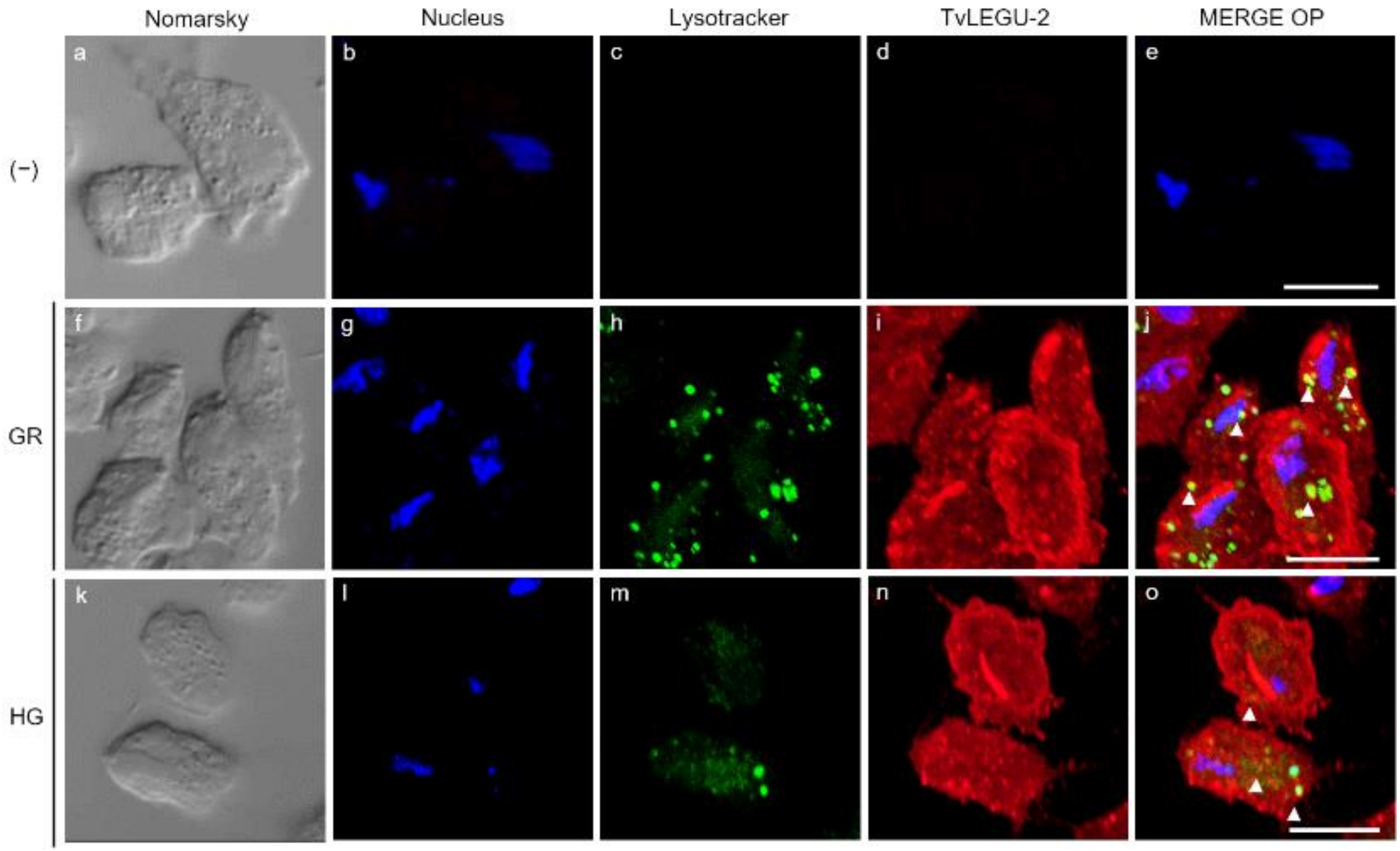

Figure S3. Orthogonal projection of the colocalization of TvLEGU-2 and LysoTracker in *T. vaginalis* under glucose restriction (GR) and high glucose (HG) conditions. IFA of permeabilized parasites under GR (f-j) and HG (k-o) conditions with Mα-TvLEGU-2pep (1:100) and LysoTracker 5 µM to label lysosomes. Negative control with PI serum or without primary antibody (a-e). TvLEGU-2 (Alexa 647, red), LysoTracker (green), and nucleus (DAPI, blue). White bar= 10 µm. White arrowhead= colocalization of TvLEGU-2 and LysoTracker.
