# Supplementary material for: Trichomonas vaginalis Legumain-2, TvLEGU-2, Is an Immunogenic Cysteine Peptidase Expressed during Trichomonal Infection
Source: Pathogens. 2024 Jan 27;13(2):119. doi: 10.3390/pathogens13020119 (PMC10892250; doi:10.3390/pathogens13020119)
Supplement: Supplementary file 1 [file pathogens-13-00119-s001.zip › Supplementary Figure S4 sent 260124.pdf]

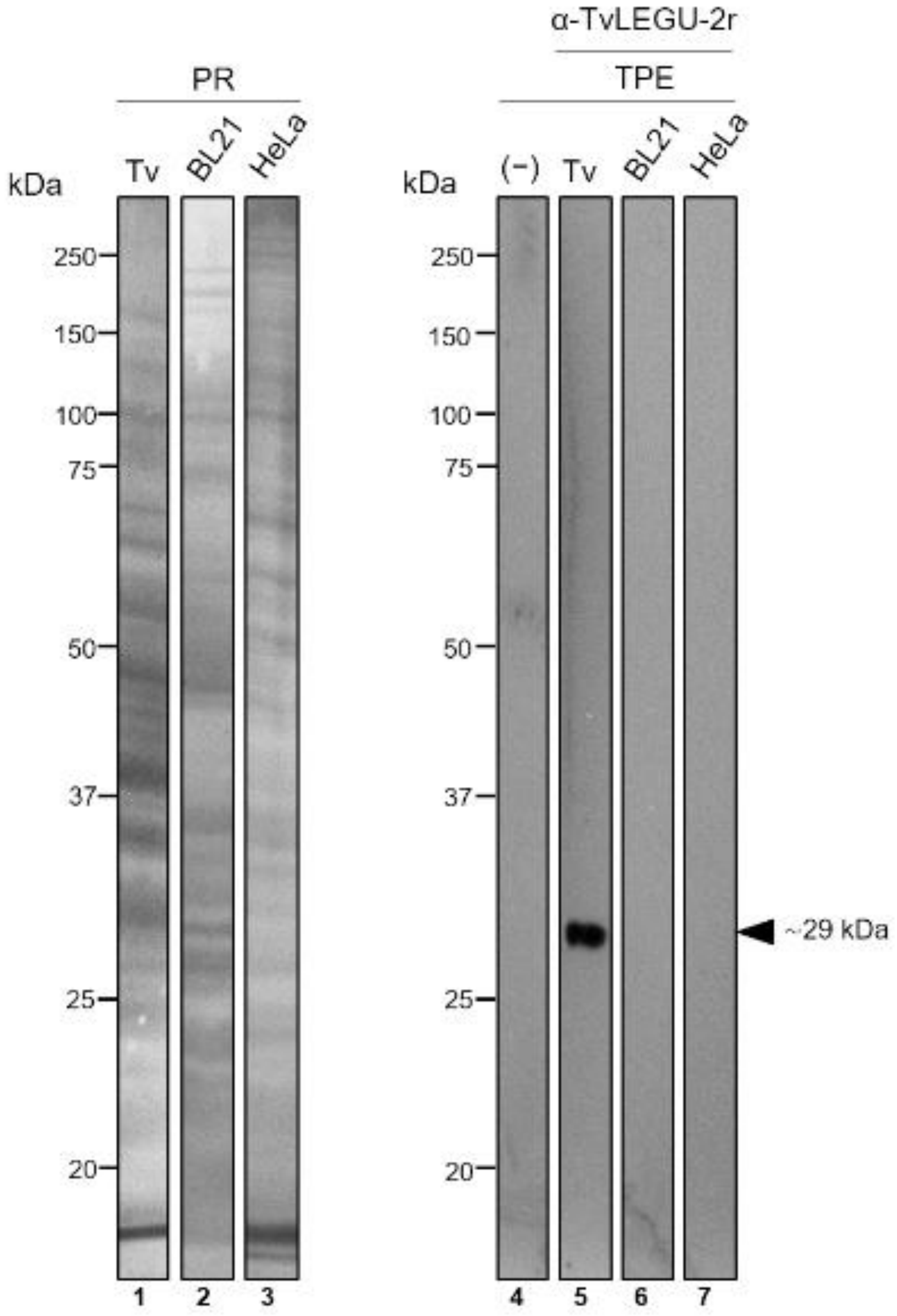

Figure S4. The anti-TvLEGU-2r antibody is specific. WB assay of total protein extracts (TPEs) of Tv, *E. coli*, and HeLa cells. Ponceau red (PR)-stained NC membranes showing the protein patterns of *T. vaginalis* (Lane 1), *E. coli* BL21 (DE3) (Lane 2), and HeLa cells (Lane 3) precipitated by TCA, separated by SDS–PAGE using 10% polyacrylamide gels, and transferred onto NC membranes. Western blot analysis of TPE from Tv (Lane 5), *E. coli* BL21 (DE3) (Lane 6), and HeLa cells (Lane 7) incubated with anti-TvLEGU-2r (1:1000 dilution) antibody. Negative controls (−) of the WB assay were performed with PI serum or without primary antibody (Lane 4). Arrowhead points to the mature protein of TvLEGU-2 (~29 kDa).
